# Supplementary material for: Functional recovery prediction during rehabilitation after rotator cuff tears by decision support system
Source: PLoS One. 2024 Mar 25;19(3):e0296984. doi: 10.1371/journal.pone.0296984 (PMC10962824; doi:10.1371/journal.pone.0296984)
Supplement: S1 Table — (PDF) [file pone.0296984.s001.pdf]

S1 Table. Rehabilitation protocol decision-making matrix (I and II assessments, points).

**I assessments (at the beginning of rehabilitation)**																					
**Criteria**	**Patients**																				
	1	2	3	4	5	6	7	8	9	10	11	12	13	14	15	16	17	18	19	20	**21**
c.4.1.	1	2	2	2	1	2	1	3	2	2	1	1	2	2	2	3	2	1	2	2	**1**
c.4.2.	2	1	2	1	2	2	2	2	1	1	2	2	1	2	1	2	2	1	2	2	**1**
c.4.3.	4	2	4	1	4	3	4	4	2	2	4	4	3	3	2	4	4	3	4	4	**1**
c.4.4.	1	2	1	1	1	1	1	1	2	1	1	1	2	1	1	2	1	1	2	1	**1**
c.4.5.	2	3	2	2	1	2	1	2	1	3	1	1	1	1	3	1	1	2	1	1	**1**
c.1.1.	1	1	2	1	2	3	3	2	3	2	3	2	3	3	4	3	3	2	2	2	**1**
c.1.2.	2	2	2	1	1	2	2	2	2	1	2	2	1	1	3	2	4	2	3	3	**4**
c.1.3.	4	2	3	2	2	3	2	2	2	3	3	4	1	5	1	5	5	1	1	5	**1**
c.1.4.	3	3	1	3	2	2	2	2	3	4	2	1	2	2	3	2	1	3	2	4	**4**
c.1.5	1	1	1	1	1	1	1	1	1	1	1	1	2	2	1	1	2	1	1	1	**2**
c.1.6.	1	2	1	2	3	4	1	3	2	4	2	3	4	2	2	3	1	2	3	4	**1**
c2.1	1	2	1	1	1	1	1	1	1	1	1	1	1	1	1	1	1	1	1	2	**2**
c.2.2.	4	3	3	4	4	3	4	3	4	3	4	4	3	3	4	3	3	4	3	3	**4**
c.2.3.	2	3	4	3	3	3	3	3	4	3	3	3	2	3	3	3	3	3	3	2	**1**
c.2.4.	1	1	2	1	2	1	2	2	1	1	1	2	2	2	1	2	2	1	1	1	**3**
c.2.5.	2	1	2	1	1	1	1	2	1	2	2	2	1	1	2	1	2	1	2	2	**1**
c3	2	2	1	2	1	2	1	3	2	1	1	1	1	2	1	1	2	2	3	1	**1**
c5	2	2	2	2	2	2	2	1	2	2	2	2	1	1	2	2	2	1	2	2	**1**
c6	1	2	3	2	3	1	4	3	2	4	2	3	2	3	2	2	4	2	3	3	**1**
**I assessments (at the end of rehabilitation)**																					
	**Patients**																				
**Criteria**	1	2	3	4	5	6	7	8	9	10	11	12	13	14	15	16	17	18	19	20	**21**
c.4.1.	1	2	2	2	1	2	1	3	2	2	1	1	2	2	2	3	2	1	2	2	**1**
c.4.2.	2	1	2	1	2	2	2	2	1	1	2	2	1	2	1	2	2	1	2	2	**1**
c.4.3.	4	2	4	1	4	3	4	4	2	2	4	4	3	3	2	4	4	3	4	4	**1**
c.4.4.	1	2	1	1	1	1	1	1	2	1	1	1	2	1	1	2	1	1	2	1	**1**
c.4.5.	2	3	2	2	1	2	1	2	1	3	1	1	1	1	3	1	1	2	1	1	**1**
c.1.1.	1	1	1	1	2	2	3	1	2	2	3	2	2	3	1	3	3	1	2	1	**1**
c.1.2.	4	2	4	2	2	2	4	4	4	2	2	4	2	1	4	4	2	2	4	4	**4**
c.1.3.	5	3	3	3	2	3	2	3	3	3	3	4	2	1	2	5	5	2	2	5	**1**
c.1.4.	4	4	3	4	4	2	3	2	2	4	4	4	3	2	3	4	4	3	4	3	**4**
c.1.5	2	2	2	1	2	2	1	2	2	1	1	1	2	2	2	1	2	1	2	2	**2**
c.1.6.	1	1	1	3	1	2	1	2	2	4	2	3	2	4	2	3	1	2	3	1	**1**
c2.1	2	2	2	2	1	1	2	1	2	2	2	2	1	1	2	1	2	1	1	2	**2**
c.2.2.	4	4	3	4	4	3	4	3	4	4	4	4	3	3	4	3	4	4	3	4	**4**
c.2.3.	1	1	1	1	2	2	2	2	1	2	2	1	1	1	1	2	1	2	2	1	**1**
c.2.4.	3	1	2	1	2	3	2	2	1	2	3	1	2	2	2	2	1	2	2	1	**3**
c.2.5.	1	1	2	1	1	1	1	1	1	2	2	1	1	1	2	1	1	1	2	1	**1**
c3	2	1	1	1	1	2	1	3	1	1	1	1	1	1	1	1	1	2	2	1	**1**
c5	2	1	2	1	2	2	2	1	1	1	1	1	1	1	2	1	1	1	2	1	**1**
c6	1	2	3	1	1	1	1	2	2	1	1	2	2	2	1	1	1	2	1	2	**1**
