# Supplementary material for: Functional recovery prediction during rehabilitation after rotator cuff tears by decision support system
Source: PLoS One. 2024 Mar 25;19(3):e0296984. doi: 10.1371/journal.pone.0296984 (PMC10962824; doi:10.1371/journal.pone.0296984)
Supplement: S2 Table — (PDF) [file pone.0296984.s002.pdf]

S2 Table. Weighted decision-making matrix during I-II assessments.

Criteria	Patients (I assessment)																				
	**1**	**2**	**3**	**4**	**5**	**6**	**7**	**8**	**9**	**10**	**11**	**12**	**13**	**14**	**15**	**16**	**17**	**18**	**19**	**20**	**21**
c.4.1.	0,0229	0,0114	0,0114	0,0114	0,0229	0,0114	0,0229	0,0000	0,0114	0,0114	0,0229	0,0229	0,0114	0,0114	0,0114	0,0000	0,0114	0,0229	0,0114	0,0114	**0,0229**
c.4.2.	0,0000	0,0115	0,0000	0,0115	0,0000	0,0000	0,0000	0,0000	0,0115	0,0115	0,0000	0,0000	0,0115	0,0000	0,0115	0,0000	0,0000	0,0115	0,0000	0,0000	**0,0115**
c.4.3.	0,0000	0,0267	0,0000	0,0400	0,0000	0,0133	0,0000	0,0000	0,0267	0,0267	0,0000	0,0000	0,0133	0,0133	0,0267	0,0000	0,0000	0,0133	0,0000	0,0000	**0,0400**
c.4.4.	0,0172	0,0000	0,0172	0,0172	0,0172	0,0172	0,0172	0,0172	0,0000	0,0172	0,0172	0,0172	0,0000	0,0172	0,0172	0,0000	0,0172	0,0172	0,0000	0,0172	**0,0172**
c.4.5.	0,0257	0,0000	0,0257	0,0257	0,0514	0,0257	0,0514	0,0257	0,0514	0,0000	0,0514	0,0514	0,0514	0,0514	0,0000	0,0514	0,0514	0,0257	0,0514	0,0514	**0,0514**
c.1.1.	0,1210	0,1210	0,0807	0,1210	0,0807	0,0403	0,0403	0,0807	0,0403	0,0807	0,0403	0,0807	0,0403	0,0403	0,0000	0,0403	0,0403	0,0807	0,0807	0,0807	**0,1210**
c.1.2.	0,0217	0,0217	0,0217	0,0000	0,0000	0,0217	0,0217	0,0217	0,0217	0,0000	0,0217	0,0217	0,0000	0,0000	0,0433	0,0217	0,0650	0,0217	0,0433	0,0433	**0,0650**
c.1.3.	0,0163	0,0488	0,0325	0,0488	0,0488	0,0325	0,0488	0,0488	0,0488	0,0325	0,0325	0,0163	0,0650	0,0000	0,0650	0,0000	0,0000	0,0650	0,0650	0,0000	**0,0650**
c.1.4.	0,0258	0,0258	0,0000	0,0258	0,0129	0,0129	0,0129	0,0129	0,0258	0,0387	0,0129	0,0000	0,0129	0,0129	0,0258	0,0129	0,0000	0,0258	0,0129	0,0387	**0,0387**
c.1.5	0,0000	0,0000	0,0000	0,0000	0,0000	0,0000	0,0000	0,0000	0,0000	0,0000	0,0000	0,0000	0,0453	0,0453	0,0000	0,0000	0,0453	0,0000	0,0000	0,0000	**0,0453**
c.1.6.	0,0222	0,0148	0,0222	0,0148	0,0074	0,0000	0,0222	0,0074	0,0148	0,0000	0,0148	0,0074	0,0000	0,0148	0,0148	0,0074	0,0222	0,0148	0,0074	0,0000	**0,0222**
c2.1	0,0000	0,0912	0,0000	0,0000	0,0000	0,0000	0,0000	0,0000	0,0000	0,0000	0,0000	0,0000	0,0000	0,0000	0,0000	0,0000	0,0000	0,0000	0,0000	0,0912	**0,0912**
c.2.2.	0,0698	0,0000	0,0000	0,0698	0,0698	0,0000	0,0698	0,0000	0,0698	0,0000	0,0698	0,0698	0,0000	0,0000	0,0698	0,0000	0,0000	0,0698	0,0000	0,0000	**0,0698**
c.2.3.	0,0181	0,0090	0,0000	0,0090	0,0090	0,0090	0,0090	0,0090	0,0000	0,0090	0,0090	0,0090	0,0181	0,0090	0,0090	0,0090	0,0090	0,0090	0,0090	0,0181	**0,0271**
c.2.4.	0,0000	0,0000	0,0082	0,0000	0,0082	0,0000	0,0082	0,0082	0,0000	0,0000	0,0000	0,0082	0,0082	0,0082	0,0000	0,0082	0,0082	0,0000	0,0000	0,0000	**0,0164**
c.2.5.	0,0000	0,0378	0,0000	0,0378	0,0378	0,0378	0,0378	0,0000	0,0378	0,0000	0,0000	0,0000	0,0378	0,0378	0,0000	0,0378	0,0000	0,0378	0,0000	0,0000	**0,0378**
c3	0,0738	0,0738	0,1476	0,0738	0,1476	0,0738	0,1476	0,0000	0,0738	0,1476	0,1476	0,1476	0,1476	0,0738	0,1476	0,1476	0,0738	0,0738	0,0000	0,1476	**0,1476**
c5	0,0000	0,0000	0,0000	0,0000	0,0000	0,0000	0,0000	0,0731	0,0000	0,0000	0,0000	0,0000	0,0731	0,0731	0,0000	0,0000	0,0000	0,0731	0,0000	0,0000	**0,0731**
c6	0,0368	0,0245	0,0123	0,0245	0,0123	0,0368	0,0000	0,0123	0,0245	0,0000	0,0245	0,0123	0,0245	0,0123	0,0245	0,0245	0,0000	0,0245	0,0123	0,0123	**0,0368**
**Total:**	**0.4712**	**0.5179**	**0.3795**	**0.5312**	**0.5260**	**0.3325**	**0.5099**	**0.3169**	**0.4583**	**0.3753**	**0.4647**	**0.4644**	**0.5605**	**0.4209**	**0.4667**	**0.3609**	**0.3440**	**0.5866**	**0.2935**	**0.5118**	**1.0000**

Criteria	**Patients (II assessment)**																				
	**1**	**2**	**3**	**4**	**5**	**6**	**7**	**8**	**9**	**10**	**11**	**12**	**13**	**14**	**15**	**16**	**17**	**18**	**19**	**20**	**21**
c.4.1.	0,0229	0,0114	0,0114	0,0114	0,0229	0,0114	0,0229	0,0000	0,0114	0,0114	0,0229	0,0229	0,0114	0,0114	0,0114	0,0000	0,0114	0,0229	0,0114	0,0114	**0,0229**
c.4.2.	0,0000	0,0115	0,0000	0,0115	0,0000	0,0000	0,0000	0,0000	0,0115	0,0115	0,0000	0,0000	0,0115	0,0000	0,0115	0,0000	0,0000	0,0115	0,0000	0,0000	**0,0115**
c.4.3.	0,0000	0,0267	0,0000	0,0400	0,0000	0,0133	0,0000	0,0000	0,0267	0,0267	0,0000	0,0000	0,0133	0,0133	0,0267	0,0000	0,0000	0,0133	0,0000	0,0000	**0,0400**
c.4.4.	0,0172	0,0000	0,0172	0,0172	0,0172	0,0172	0,0172	0,0172	0,0000	0,0172	0,0172	0,0172	0,0000	0,0172	0,0172	0,0000	0,0172	0,0172	0,0000	0,0172	**0,0172**
c.4.5.	0,0257	0,0000	0,0257	0,0257	0,0514	0,0257	0,0514	0,0257	0,0514	0,0000	0,0514	0,0514	0,0514	0,0514	0,0000	0,0514	0,0514	0,0257	0,0514	0,0514	**0,0514**
c.1.1.	0,1210	0,1210	0,1210	0,1210	0,0605	0,0605	0,0000	0,1210	0,0605	0,0605	0,0000	0,0605	0,0605	0,0000	0,1210	0,0000	0,0000	0,1210	0,0605	0,1210	**0,1210**
c.1.2.	0,0650	0,0217	0,0650	0,0217	0,0217	0,0217	0,0650	0,0650	0,0650	0,0217	0,0217	0,0650	0,0217	0,0000	0,0650	0,0650	0,0217	0,0217	0,0650	0,0650	**0,0650**
c.1.3.	0,0000	0,0325	0,0325	0,0325	0,0488	0,0325	0,0488	0,0325	0,0325	0,0325	0,0325	0,0163	0,0488	0,0650	0,0488	0,0000	0,0000	0,0488	0,0488	0,0000	**0,0650**
c.1.4.	0,0387	0,0387	0,0193	0,0387	0,0387	0,0000	0,0193	0,0000	0,0000	0,0387	0,0387	0,0387	0,0193	0,0000	0,0193	0,0387	0,0387	0,0193	0,0387	0,0193	**0,0387**
c.1.5	0,0453	0,0453	0,0453	0,0000	0,0453	0,0453	0,0000	0,0453	0,0453	0,0000	0,0000	0,0000	0,0453	0,0453	0,0453	0,0000	0,0453	0,0000	0,0453	0,0453	**0,0453**
c.1.6.	0,0222	0,0222	0,0222	0,0074	0,0222	0,0148	0,0222	0,0148	0,0148	0,0000	0,0148	0,0074	0,0148	0,0000	0,0148	0,0074	0,0222	0,0148	0,0074	0,0222	**0,0222**
c2.1	0,0912	0,0912	0,0912	0,0912	0,0000	0,0000	0,0912	0,0000	0,0912	0,0912	0,0912	0,0912	0,0000	0,0000	0,0912	0,0000	0,0912	0,0000	0,0000	0,0912	**0,0912**
c.2.2.	0,0698	0,0698	0,0000	0,0698	0,0698	0,0000	0,0698	0,0000	0,0698	0,0698	0,0698	0,0698	0,0000	0,0000	0,0698	0,0000	0,0698	0,0698	0,0000	0,0698	**0,0698**
c.2.3.	0,0271	0,0271	0,0271	0,0271	0,0000	0,0000	0,0000	0,0000	0,0271	0,0000	0,0000	0,0271	0,0271	0,0271	0,0271	0,0000	0,0271	0,0000	0,0000	0,0271	**0,0271**
c.2.4.	0,0164	0,0000	0,0082	0,0000	0,0082	0,0164	0,0082	0,0082	0,0000	0,0082	0,0164	0,0000	0,0082	0,0082	0,0082	0,0082	0,0000	0,0082	0,0082	0,0000	**0,0164**
c.2.5.	0,0378	0,0378	0,0000	0,0378	0,0378	0,0378	0,0378	0,0378	0,0378	0,0000	0,0000	0,0378	0,0378	0,0378	0,0000	0,0378	0,0378	0,0378	0,0000	0,0378	**0,0378**
c3	0,0738	0,1476	0,1476	0,1476	0,1476	0,0738	0,1476	0,0000	0,1476	0,1476	0,1476	0,1476	0,1476	0,1476	0,1476	0,1476	0,1476	0,0738	0,0738	0,1476	**0,1476**
c5	0,0000	0,0731	0,0000	0,0731	0,0000	0,0000	0,0000	0,0731	0,0731	0,0731	0,0731	0,0731	0,0731	0,0731	0,0000	0,0731	0,0731	0,0731	0,0000	0,0731	**0,0731**
c6	0,0368	0,0184	0,0000	0,0368	0,0368	0,0368	0,0368	0,0184	0,0184	0,0368	0,0368	0,0184	0,0184	0,0184	0,0368	0,0368	0,0368	0,0184	0,0368	0,0184	**0,0368**
**Total:**	**0.7109**	**0.7959**	**0.6338**	**0.8104**	**0.6288**	**0.4073**	**0.6382**	**0.4590**	**0.7841**	**0.6468**	**0.6341**	**0.7443**	**0.6102**	**0.5159**	**0.7617**	**0.4660**	**0.6912**	**0.5973**	**0.4473**	**0.8179**	**1.0000**
