# Supplementary material for: Functional recovery prediction during rehabilitation after rotator cuff tears by decision support system
Source: PLoS One. 2024 Mar 25;19(3):e0296984. doi: 10.1371/journal.pone.0296984 (PMC10962824; doi:10.1371/journal.pone.0296984)
Supplement: Attachment — Submitted filename: Respond to Editors.pdf [file pone.0296984.s004.pdf]

ANSWER to EDITORS

Reason to Reject PONE – D-23-28958

We recognize that you obtained ethical approval from your local ethics committee after you began the study: your ethics approval document is dated May 25, 2021, while participants were recruited beginning on May 21, 2021. For human subjects research, PLOS ONE requires that authors obtain ethics committee approval before beginning the study, and we do not accept retrospective ethical approval.

We are very sorry that a technical, human error was made when entering the recruitment term. We have corrected and resubmitted the publication and hope for its scientific value.
