# Supplementary material for: Functional recovery prediction during rehabilitation after rotator cuff tears by decision support system
Source: PLoS One. 2024 Mar 25;19(3):e0296984. doi: 10.1371/journal.pone.0296984 (PMC10962824; doi:10.1371/journal.pone.0296984)
Supplement: Attachment — Submitted filename: Functional recovery prediction during rehabilitation after cuff tears by decision support.sohani comments.pdf [file pone.0296984.s005.pdf]

PLOS ONE

Functional recovery prediction during rehabilitation after cuff tears by decision support system

--Manuscript Draft--

**Manuscript Number:**	PONE-D-23-31967
**Article Type:**	Research Article
**Full Title:**	Functional recovery prediction during rehabilitation after cuff tears by decision support system
**Short Title:**	Functional recovery prediction
**Corresponding Author:**	Kristina Daunoraviciene, PhD<br>Vilnius Gediminas Technical University: Vilniaus Gedimino Technikos Universitetas<br>Vilnius, Vilniaus m. sav. LITHUANIA
**Keywords:**	Rotator cuff tears, Rehabilitation, Functional recovery, Evaluation, MCDM methodology
**Abstract:**	Background: Today's rehabilitation decision-making still relies on conventional methods and different specific targeted rehabilitation protocols. Our study focuses on the decision support system for rehabilitation after rotator cuff tears (RCT), where a multicriteria decision-making framework (MCDM) is applied for the evaluation of the patient physical and functional state.<br>Objective: To identify factors that affect recovery outcomes and to develop a decision support system methodology for predicting functional recovery outcomes at early rehabilitation after RC repair.<br>Methods: 12 rehabilitation experts were involved in the case study of the design, calibration, and evaluation of a rehabilitation protocol based on the proposed decision support system constructed applying the MCDM framework.<br>Results: Final results revealed the following: (1) at the beginning of rehabilitation, 90% of patients had Severe and Moderate impairment and functional problems in daily life activities and were classified as II-III classes; (2) the effectiveness of rehabilitation measures is indisputable and at the end of rehabilitation was detected a significant improvement in shoulder motor function, a decrease of pain and reduction of disability. 80% of patients have Moderate and Mild impairment and functional problems in daily life activities and were classified as III and IV classes.<br>Conclusions: The proposed decision support system showed that for expert-selected main criteria, the applied MCDM methods perfectly contribute to the criterion weighting and the final distinguishment into impairment classes.
**Order of Authors:**	Aušra Adomavičienė, Assoc. Prof. Dr.<br>Kristina Daunoraviciene, Prof. Dr.<br>Girūta Kazakevičiūtė-Januškevičienė, Assoc. Prof. Dr.<br>Romualdas Baušys, Prof. habil. Dr.
**Additional Information:**	
**Question**	**Response**
**Financial Disclosure**<br><br>Enter a financial disclosure statement that describes the sources of funding for the work included in this submission. Review the submission guidelines for detailed requirements. View published research articles from *PLOS ONE* for specific examples.	The author(s) received no specific funding for this work.

Powered by Editorial Manager® and ProduXion Manager® from Aries Systems Corporation

This statement is required for submission and **will appear in the published article** if the submission is accepted. Please make sure it is accurate.<br><br>**Funded studies**<br>Enter a statement with the following details:<br>• Initials of the authors who received each award<br>• Grant numbers awarded to each author<br>• The full name of each funder<br>• URL of each funder website<br>• Did the sponsors or funders play any role in the study design, data collection and analysis, decision to publish, or preparation of the manuscript?<br><br>Did you receive funding for this work?	
**Competing Interests**<br><br>Use the instructions below to enter a competing interest statement for this submission. On behalf of all authors, disclose any competing interests that could be perceived to bias this work—acknowledging all financial support and any other relevant financial or non-financial competing interests.<br><br>This statement is required for submission and **will appear in the published article** if the submission is accepted. Please make sure it is accurate and that any funding sources listed in your Funding Information later in the submission form are also declared in your Financial Disclosure statement.<br><br>View published research articles from *PLOS ONE* for specific examples.	The authors have declared that no competing interests exist.

**NO authors have competing interests**<br><br>Enter: *The authors have declared that no competing interests exist.*<br><br>**Authors with competing interests**<br><br>Enter competing interest details beginning with this statement:<br><br>*I have read the journal's policy and the authors of this manuscript have the following competing interests: [insert competing interests here]*<br><br>* typeset	
**Ethics Statement**<br><br>Enter an ethics statement for this submission. This statement is required if the study involved:<br><br>• Human participants<br>• Human specimens or tissue<br>• Vertebrate animals or cephalopods<br>• Vertebrate embryos or tissues<br>• Field research<br><br>Write "N/A" if the submission does not require an ethics statement.<br><br>General guidance is provided below. Consult the submission guidelines for detailed instructions. **Make sure that all information entered here is included in the Methods section of the manuscript.**	Data collected according Ethical Protocol No KFVDSGS2021 provided by Vilnius Regional Biomedical Research Ethics Committee. Written consent obtained.

**Format for specific study types**<br><br>**Human Subject Research (involving human participants and/or tissue)**<br>• Give the name of the institutional review board or ethics committee that approved the study<br>• Include the approval number and/or a statement indicating approval of this research<br>• Indicate the form of consent obtained (written/oral) or the reason that consent was not obtained (e.g. the data were analyzed anonymously)<br><br>**Animal Research (involving vertebrate animals, embryos or tissues)**<br>• Provide the name of the Institutional Animal Care and Use Committee (IACUC) or other relevant ethics board that reviewed the study protocol, and indicate whether they approved this research or granted a formal waiver of ethical approval<br>• Include an approval number if one was obtained<br>• If the study involved *non-human primates*, add *additional details* about animal welfare and steps taken to ameliorate suffering<br>• If anesthesia, euthanasia, or any kind of animal sacrifice is part of the study, include briefly which substances and/or methods were applied<br><br>**Field Research**<br><br>Include the following details if this study involves the collection of plant, animal, or other materials from a natural setting:<br>• Field permit number<br>• Name of the institution or relevant body that granted permission	
**Data Availability**<br><br>Authors are required to make all data underlying the findings described fully available, without restriction, and from the time of publication. PLOS allows rare exceptions to address legal and ethical concerns. See the PLOS Data Policy and FAQ for detailed information.	Yes - all data are fully available without restriction

A Data Availability Statement describing where the data can be found is required at submission. Your answers to this question constitute the Data Availability Statement and **will be published in the article**, if accepted.<br><br>**Important:** Stating 'data available on request from the author' is not sufficient. If your data are only available upon request, select 'No' for the first question and explain your exceptional situation in the text box.<br><br>Do the authors confirm that all data underlying the findings described in their manuscript are fully available without restriction?	
**Describe where the data may be found in full sentences. If you are copying our sample text, replace any instances of XXX with the appropriate details.**<br><br>• If the data are **held or will be held in a public repository**, include URLs, accession numbers or DOIs. If this information will only be available after acceptance, indicate this by ticking the box below. For example: *All XXX files are available from the XXX database (accession number(s) XXX, XXX.*).<br>• If the data are all contained **within the manuscript and/or Supporting Information files**, enter the following: *All relevant data are within the manuscript and its Supporting Information files.*<br>• If neither of these applies but you are able to provide **details of access elsewhere**, with or without limitations, please do so. For example:<br><br>*Data cannot be shared publicly because of [XXX]. Data are available from the XXX Institutional Data Access / Ethics Committee (contact via XXX) for researchers who meet the criteria for access to confidential data.*<br><br>*The data underlying the results presented in the study are available from (include the name of the third party*	All relevant data are within the manuscript and its Supporting Information files.

and contact information or URL).<br>• This text is appropriate if the data are owned by a third party and authors do not have permission to share the data.<br><br>* typeset	
Additional data availability information:	

Powered by Editorial Manager® and ProduXion Manager® from Aries Systems Corporation

Manuscript

Click here to access/download;Manuscript;FunctionalRecoveryPrediction.do

Functional recovery prediction during rehabilitation after rotator cuff tears by decision support system

Aušra Adomavičienė[1], Kristina Daunoravičienė[2*], Girūta Kazakevičiūtė-Januškevičienė[3], Romualdas Baušys [3]

[1] Department of Rehabilitation, Physical and Sports Medicine, Faculty of Medicine, Vilnius University, Vilnius, Lithuania

[2] Department of Biomechanical Engineering, Vilnius Gediminas technical University, Vilnius, Lithuania

[3] Department of Graphical Systems, Faculty of Fundamental Sciences, Vilnius Gediminas technical University, Vilnius, Lithuania

* Corresponding author

E-mail: kristina.daunoraviciene@vilniustech.lt

These authors contributed equally to this work.

These authors also contributed equally to this work

Abstract

Background: Today's rehabilitation decision-making still relies on conventional methods and different specific targeted rehabilitation protocols. Our study focuses on the decision support system for rehabilitation after rotator cuff tears (RCT), where a multicriteria decision-making framework (MCDM) is applied for the evaluation of the patient physical and functional state.

Objective: To identify factors that affect recovery outcomes and to develop a decision support system methodology for predicting functional recovery outcomes at early rehabilitation after RC repair.

Methods: 12 rehabilitation experts were involved in the case study of the design, calibration, and evaluation of a rehabilitation protocol based on the proposed decision support system constructed applying the MCDM framework.

Results: Final results revealed the following: (1) at the beginning of rehabilitation, 90% of patients had Severe and Moderate impairment and functional problems in daily life activities and were classified as II-III classes; (2) the effectiveness of rehabilitation measures is indisputable and at the end of rehabilitation was detected a significant improvement in shoulder motor function, a decrease of pain and reduction of disability. 80% of patients have Moderate and Mild impairment and functional problems in daily life activities and were classified as III and IV classes.

Conclusions: The proposed decision support system showed that for expert-selected main criteria, the applied MCDM methods perfectly contribute to the criterion weighting and the final distinguishment into impairment classes.

Keywords: Rotator cuff tears, Rehabilitation, Functional recovery, Evaluation, MCDM methodology

1. Introdu[illegible]on

Rotator cuff tears (RCTs) are a very common condition that often causes disability, is a common cause of shoulder pain, and can result in weakness, shoulder instability, and limitation of daily activities (work and sports). The symptomatic illness affects between 4% and 32% of patients with RC tears. Successful management of RC depends on adequate rehabilitation, whether non-surgical or post-surgical. The main goals of postoperative rehabilitation after RC repair are to protect the healing process and prevent joint stiffness and muscle atrophy in parallel. However, there are not many evidence-based studies regarding postoperative rehabilitation protocols. Some authors support a traditional-conservative program [1], while others claim that both protective and accelerated rehabilitation protocols, based on the individual case, reduce the risk of postoperative stiffness without compromising the final result [2]. There are many factors that directly affect rehabilitation. First of all, these are medical factors related to tendon rupture, such as surgical approach, quality of the tendon, localization and configuration of the rupture, and etiology of the rupture (degenerative or traumatic). Also other influential factors are sociodemographic, functional and rehabilitation programs and measures [3]. Rehabilitation after RC repair begins with close communication between the patient and the rehabilitation team as well as the surgeon and must continue throughout the recovery process. The physical therapist must gather all the information and details that are useful to create an appropriate and successful rehabilitation protocol. These protocols vary between providers and depend on the selection of progression time and therapeutic exercise. Regardless of whether a conservative or moderate rehabilitation protocol is chosen, the primary goals should always be the same, which are to maintain repair, reduce tendon stress and pain, and expedite the patient's return to the patient's previous life activities.

Previous reviews [4, 5] show that the evaluation of rehabilitation protocols and measures after RC tears has received insufficient attention. Usually, the efficiency, intensity and duration of

conventional rehabilitation measures are examined, or the pain syndrome, the protocol of measures to reduce shoulder stiffness and techniques that increase the functional recovery of the shoulder and reduce disability after rehabilitation are analyzed separately. Factors that contribute to successful or unsuccessful rehabilitation outcomes are typically analyzed: shoulder motor function (ROM, muscle strength, and endurance), shoulder mobility and stiffness, pain syndrome, functional problems, activities of daily living limitations, and disability. Many risk factors for functional recovery and reduction of rehabilitation efficiency are known, which usually decide postoperative stiffness, such as adhesive capsulitis, concomitant labral repair, single tendon rotator cuff repair, coexisting calcific tendinitis, pain syndrome and shoulder disability [6]. To avoid all these consequences, an earlier rehabilitation team should start with a clear and detailed view of complex individual patient functional and physical state analyses through prognostic decision-making assistance.

So, what is the best rehabilitation protocol?! And what about the one that is based on precise and competent decisions and directed purposefully and individually to the dysfunction and its causes? Decision-making is the process of making choices by identifying a decision, gathering information, and weighing alternative resolutions. Decision theories are based on mathematical, prognostic and probabilistic methods, and during the last century, they have been proposed in many different fields. The theory of multi-criteria decision-making (MCDM) was introduced in the second half of the 20th century and helped the decision-maker to solve problems where interacting criteria are involved and need to be evaluated. MCDM is one of the most widely used decision methodologies in various fields such as energy and environment, business, economy, material selection, computer software selection, etc. [7, 8]. The application of MCDM in biomedical engineering and healthcare is not a new approach and is very useful for medical professionals, patients, engineers and others in the field [9, 10]. However, in recent years, an effective methodology in medicine MCDM is not yet in rehabilitation, but in related medical

fields such as oncology, when choosing a treatment strategy (operative or conservative treatment), drug treatment algorithms are based on MCDM [11].

Functional assessment in rehabilitation is performed by a team, and various methods are used for both assessment and protocol implementation (main tools, methods, scales – the most recent and most effective). However, until now, in rehabilitation, only attempts have been made to apply prognostic methods, and multifactorial analysis methods, based on which it would be possible to optimally create a rehabilitation protocol and select rehab measures after a very detailed analysis of various factors that determine the success of recovery and effective rehab protocol.

Today's decision-making in rehabilitation still relies on routine methods and different specific targeted rehabilitation protocols. Our study is focused on rehabilitation after Rotator cuff tears (RCT), where decision-making is carried out according to numerous rehabilitation protocols for the management of RC disease that are based primarily on clinical experience and expert opinion. MCDMs, including multi-rule decision-making, multi-objective decision-making, and multi-attribute decision-making, are used by clinical doctors to analyze healthcare problems from multiple perspectives [9, 11]. In practical healthcare cases, there are many critical parameters (criteria) that can directly or indirectly affect the consequences of different decisions. Semi-structured and unstructured decision-making issues involve multiple criteria (or objectives) that may conflict with each other [11]. Thus, the use of MCDM is a promising source of practical solutions for such problems. The use of multi-criteria methods is important because by using appropriate methods to evaluate their weights (SWARA) [7], it is possible to evaluate the superiority of the criteria in relation to each other. MCDM can be defined as the decision support/assistance in decision-making by assessing the patient's health state, functional and physical capabilities and monitoring the effectiveness and recovery of different functions at the beginning of the rehabilitation program after RC repair.

Therefore, in this paper, an MCDM-based evaluation methodology was developed and tested on the problem of the best recovery way in Rehabilitation for RC pathology.

We hypothesized that MCDM can help a rehabilitation specialist to clarify detailed rehabilitation protocol for each patient after RCT according to the patient's individual physical, functional and emotional state, postoperative risk and needs. In other words, to select criteria from many criteria and, using multifactorial methods, gradually refine those that will help to decide on the most optimal rehabilitation protocol. Therefore, **the objective** of the study is to identify factors that affect recovery outcomes and to develop a decision-support methodology for predicting functional recovery outcomes at early rehabilitation after RC repair.

The second chapter is dedicated to the analysis of related state of the art scientific works. An analysis of the methodology used for evaluating successful rehabilitation (classical and MCDM) was performed. The third chapter describes the methodology used and applied in our work for a particular selected case. And finally, the fourth and fifth chapters present the obtained results and discussion statements.

2. Analysis of works related to the topic

Rehabilitation protocols after RCT are designed to protect recovery in the immediate postoperative period, as well as to reduce postoperative stiffness and muscle atrophy and to reduce pain and functional problems. Recent studies have shown mixed results for postoperative treatment modalities and rehabilitation protocols: conservative and accelerated rehabilitation protocols did not show any significant difference, and early passive range of motion (ROM) after RC repair demonstrated reduced postoperative stiffness and improved functionality. Researchers reported exercising effectiveness on functional recovery and provided comparisons of different postoperative rehabilitation: a land-based program with or without aquatic therapy, individualized physical therapy plus exercise at home, an inpatient outpatient rehabilitation

program, progressive weight-bearing rehabilitation, home exercise instruction, etc. All the rehabilitation protocols and treatmens presented in Table 1 are primarily related to the main symptoms accompanying patients after RC tears and how they are reduced.

The functional recovery after RCT and rehabilitation outcomes are influenced not only by the different factors associated with RC pathology but also by complex and individually assigned rehabilitation measures: intensity and duration, whether it corresponds to the patient's individual physical and psycho-emotional characteristics, tolerance of physical load, the performance of independent tasks, physical activity in daily living activities.

Table 1. Various treatment and rehabilitation protocols after RCT

No.	Rehabilitation protocol/treatment methods	Targeted outcome	Author (Publication Year)
1.	Early rehabilitation protocols	Repair integrity, shoulder functional recovery, pain reduction/ improved quality of life	Berton, A. et al. (2021); Kathryn Fahy (2022); Jennifer C. Seida (2010); Roe Y. et all. (2013)
2.	A land-based program with or without aquatic therapy	Reduced pain and restored shoulder function	Byung-Su Kim (2021); Huihui Wang et all. (2022);
3.	An inpatient rehabilitation program	Reduced pain and restored shoulder function	Burak Altintas (2020); Balraj S.Jagdev (2022);
4.	An outpatient rehabilitation program/ home exercise instruction/ early return to daily life activities	Reduced pain and restored shoulder function	Jennifer C. Seida (2010); Burak Altintas et all. (2020); Maryam Daghiani (2022); Byung-Su Kim (2021)
5.	Progressive weight-bearing rehabilitation	Significant reduction in pain	Balraj S.Jagdev (2022); Yi, D et all. (2021); Robert L. et all. (2020); Burak Altintas (2020); Patrick Kennedy (2019)
6.	Postoperative nonsteroidal anti-inflammatory drugs during the first week after surgery	Pain control and reduction in 2-3 months after RC tears repair	Maryam Daghiani (2022); Laurent Audige (2021)

Different functional recovery factors are evaluated in rehabilitation, and, in fact, although they are all important, their influence on functional status depends on many circumstances. Scientists and medical professionals have identified the most important, most influencing factors after RC tears and recommend that they be closely monitored (Table 2).

Table 2. Functional recovery influencing factors in order of importance

No	Influencing factor	Evaluation methods and instruments	Rehabilitation protocol/treatment methods	Author (Publication Year)
1.	Shoulder pain and dysfunction	Visual Analog Scale for Pain (VAS)	Anti-inflammatory drugs, comfortable hand positions, Various exercises, Massages and Physiotherapeutic measures	Robert L. et. all. (2020); B.G.Lee et all. (2012); Yi, D et all. (2021); Adomavičienė et all. (2021)
2.	Shoulder functional parameters	Patient-reported questionnaire, ROMs measurement, Apley scratch test, manual muscle testing scale, spring scale, dynamometry, DASH		Zhengyu Sun (2018); Wong, W.K. (2020). Nitin B.Jain (2018) Byung-Su Kim (2021) S. Hajivandi (2021)
3.	Psychoemotional state: anxiety and depression; sleep restriction	Psychological distress and patient-reported subjective well-being	Pain relief methods, inpatient rehabilitation program, inclusion in daily life activities	Joo Hyun Park (2021); O.Nikolaidou (2017)
4.	Age	Birth data	Inclusion and participation in daily life activities	K.Saito et all. (2021); Z.Wani et all (2016); Xiao L et all. (2012); Laurent Audige (2021); Luke (2007)
5.	Duration of symptoms	Pain syndrome, shoulder stiffness and disability	Exercises and training programs, Massages and Physiotherapeutic measures	Jennifer C. Seida (2010); Luke (2007).

*NA stays for not applicable.

RC tear is one of the major causes of shoulder pain and dysfunction and is usually classified as nociceptive pain [12]. Most of the scientists are invoked on individual factors of the pain, which have an impact on different aspects of recovery: the duration of pain must be defined in days, weeks and months, nature of pain accurately assessed by qualifying adjectives: intensive, intermittent, sharp, burning and etc., the indication of the causes of the intensification of pain (in movements, a certain position, exercising) or the presence of dynamic pain (during movements) or causes of pain reduction (in movements, position, exercising, after therapies) [13, 14]. In contrast, a lot of studies highlight the potential importance of accounting for concomitant rehabilitation measures as a predictor of early postoperative pain intensity outcomes and successful pain control protocol, considering the reduced motor function of the shoulder, arm and hand and increased disability after RC tears as consequences a long-term effect of pain,

immobility and muscle stiffness, shoulder instability and limitation of daily life activities [15, 16]. Many researchers claim that there is a direct relationship between the patient's psycho-emotional state, expectations, the presence of anxiety and the results of rehabilitation [17, 18]. Sleep disorders are commonly seen in patients with RC tears, and after repair, there is an increase in the quality of sleep with a parallel improvement in shoulder functions. Consistent improvements in sleep quality are observed just in the 6 months after RC repair and are significantly related to physical recovery and decreased anxiety [17]. Age is one of the dominant factors in RC tear and also for recovery mechanisms [18, 21]. In general, RC tears are more common in the elderly than in younger patients, being chronic or acute and chronic and usually secondary to tendon degeneration. The literature shows an increasing prevalence of RC tears in the elderly, with a mean age of 60.2 years (range, 23-81 years) [19]. Some studies stated that the duration of symptoms greater than 1 year is one of the main negative prognostic factors for successful recovery [20]. A substantial percentage of patients who experienced symptoms for less than 3 months in duration reported lasting improvement [21, 22].

The further, the more medical professionals agree that functional assessment of the patient and data collection is a routine part of their work, and the need for an additional auxiliary instrument arises [23-25]. Such an additional tool as an algorithm would help indicate the main guidelines, the main criteria for the creation of an individual patient's rehabilitation program.

3. Methodology

3.1. Framework of the methodology

This section demonstrates the framework of the methodology for the identification of the criteria, selection of a rehabilitation protocol after RC tears, and classification / grouping of patients into

appropriate classes according to the success of rehabilitation. To solve the problem of our study, i.e. to make the optimal decision, the main steps for multi-criteria decision-making methodology were included: (1) identification of the problem using routine functional assessment methods in rehabilitation, discussion of factors influencing recovery and determination of their importance during the expert discussion, (2) definition of criteria and sub-criteria and their weights evaluation, (3) data processing using normalization and MCDM methods for indicators aggregation (4). Figure 1 represents the methodological steps of our study.

Fig. 1. Methodical steps of the workflow

3.2. Research team

12 rehabilitation specialists participated as experts in the experimental study: 2 PRM doctors with >15 years of rehabilitation experience, 6 physiotherapists and 4 occupational therapists with 15-10 years of experience in rehabilitation with RC pathology. All experts have a scientific degree (PhD) and are active scientists and researchers.

3.3. Participants

Problem definition starts with a patient selection due to inclusion and exclusion criteria. Inclusion criteria: (1) ≥ 40 years old, (2) with a full-thickness tear of the rotator cuff muscles, (3) 6-7 weeks after surgery, (4) underwent surgery for the first time, (5) voluntary agreement participate in the study.

Exclusion criterion: (1) RC tear retraction and repeated shoulder surgery, (2) co-morbidities leading to shoulder function; (3) >8 weeks after surgery.

24 patients with surgical recurrence of RC undergoing rehabilitation in our outpatient department were enrolled in a prospective cohort clinical trial. 5-6 weeks after surgery, the participants began

participating in 2 weeks of conventional outpatient rehabilitation. 4 patients (16.6%) did not complete the rehabilitation program due to worsening health (pain, high blood pressure, heart rate problems and intolerance of physical load). In total, 20 of 24 patients (83.3%) completed the study and were included in the analysis of final data. The assessment data was collected, scored and entered into a database by a study coordinator. The patient's consent to participate in the study was signed after informing the patient about all the details of the study. The study was approved by the Vilnius Regional Bioethics Committee and registered as a Clinical trial. The recruitment period for this study from 21.06.2021 to 20.12.2022.

3.4. Different functional factors selection

In this section, a complete theoretical model of the selection of different functional and physical state factors for a rehabilitation protocol is proposed. However, those are not individual health condition restrictions (factors of functional, physical and psycho-emotional state) compatible with the principles of rehabilitation outcomes but rather an integrated choice suitable for subsequent protocol creation for each patient. Our proposed protocol is a multi-criteria analysis system structured according to two main levels: the patient's physical-functional state and psycho-emotional state (Figure 2). At the same time, we emphasize that the usual rehabilitation program (duration and intensity) and the measures applied to patients after RC tears meet the requirements of the national rehabilitation medical regulation.

Fig. 2 Structure of proposed multicriteria system

The study was started after evaluating practically all possible selection criteria of patients after RC tears, functional and psycho-emotional condition, and factors related to RC tears and socio-demographic characteristics. 10 independent qualified experts related to RC pathology

rehabilitation decided the choice and importance of the criteria. Their opinion is highly based on the previous works' reports and their own practice. During the deliberations, the assessment of items / factors such as gender, socio-demographic state (education, employment and residence), dominated/not dominated arm and sports or physical recreation activities were excluded because they do not have a significant impact on the rehabilitation outcomes. It was unanimously agreed that the most important criterion of successful rehabilitation and functional recovery is the Pain syndrome (Intensity and duration of pain, nature of pain, causes of the intensification of pain, Intensity, nature and duration of dynamic pain ($c1$). The second important criterion is Shoulder motor function and disability (Shoulder ROM, muscle strength, hand grip strength, dexterity and functioning in daily life activities) (c2). It was found that most studies confirm that psycho-emotional factors such as anxiety, depression (c3), sleep restriction (c7) and fear of movements (c5) have a significant influence on the level of disability and pain experienced by patients before surgery. However, there is a significant improvement in postoperative pain and shoulder functional level, even in the presence of significant psychosocial impairment. While sociodemographic and injury related characteristics (c4) should be given the highest priority. These factors and characteristics (age, causes of RC pathology / injury, affected muscle, time since surgery / rehabilitation and symptoms duration) directly determine the treatment strategy (surgery or conservative treatment and rehabilitation outcomes). Patients after RC tears' physical recovery and psycho-emotional well-being are related to the presence of sleep restrictions (c6) – which are also considered a serious influencer for healing and functional recovery. The last group of criteria includes Healthcare organization and routine after RC tears - Rehabilitation measures (c7), Exercising intensity and duration (c8), Home activities in daily life (c10) and Medicines (c9). These criteria characteristics can help clinical staff to select recovery protocol and achieve successful rehabilitation outcomes and also identify patients who are prone to re-tear early after RCT repair and develop targeted prevention and treatment strategies for modifiable risk factors.

Criteria were sorted out based on the expert's qualifications and experience related to the rehabilitation after RC tears. According to experts, 3 groups of criteria were singled out: (I) Physical and functional condition, (II) Psycho-emotional condition, and (III) Healthcare organization and routine, and were ranked according to importance (Table 3).

Table 3. Criteria ($c1 - c10$) sorted by relevance.

No	Min/max	Criteria title	Descriptive parameters / Problem considered	Author (Publication Year)
**c1**	Min	Pain syndrome	Intensity and duration of pain, Nature of pain; Intensity, nature and duration of dynamic pain	Eivind Inderhaug (2018); Burak Altintas (2020); Balraj S.Jagdev (2022); Patrick Kennedy (2019); J.Tangtiphaiboontana (2021); Yi. D (2021); Laurent Audige (2021); Robert L.Brochin (2020);
**c2**	Max	Shoulder motor function and disability	Shoulder ROM, muscle strength, dexterity of movements, Functioning and disability in daily activities	Patrick Kennedy (2019) Robert L.Brochin (2020) Laurent Audige (2021) Nitin B.Jain (2018) Byung-Su Kim (2021) Shaahin Hajivandi (2021)
**c3**	Min	Anxiety / Depression	Presence of anxiety / depression symptoms	Joo Hyun Park (2021) Wong, W.K (2020) A.Adomavičienė (2021) Patrick Kennedy (2019) (Yngve Roe, 2013)
**c4**	Min	Sociodemographic and with injury related characteristics	Age, causes of injury, time since surgery intervention, affected muscles	Patrick Kennedy (2019) Huihui Wang (2022) Z.Wani (2016) (Yngve Roe, 2013)
**c5**	Min	Fear of movements	Fear of shoulder movements	Huihui Wang (2022) Eivind Inderhaug (2018) Patrick Kennedy (2019)
**c6**	Min	Sleep restriction	Presence of sleep restriction	Kyle N. Kunze (2020) Patrick Kennedy (2019) (Yngve Roe, 2013)
**c7**	Max	Rehabilitation program measures	Conventional complex rehabilitation program	Maryam Daghiani (2022) Laurent Audige (2021) Robert L.Brochin (2020)
**c8**	Max	Rehab program intensity and duration	2-2.5 weeks, 3.5-4 h/day, individually designed program	Robert L.Brochin (2020) Nitin B.Jain (2018) Byung-Su Kim (2021) Huihui Wang (2022)
**c9**	Min	Medicines	Use of medication (analgesics, anti-inflammatory drugs)	Patrick Kennedy (2019) Maryam Daghiani (2022) Laurent Audige (2021)

**c10**	Max	Home activities in daily life and exercising intensity	Intensity of exercising and ADL activity at home	Maryam Daghiani (2022)<br>Byung-Su Kim (2021)<br>Huihui Wang (2022)<br>Eivind Inderhaug (2018)

Also, each group of criteria was separated into sub-criteria, and they were ranked according to importance, expressed as a percentage and numbers. Experts ranked the factors in order of importance; therefore, their views are assumed to be consistent. Based on the criteria and the ranking of sub-criteria by importance presented in Table 4, a pairwise assessment of the relative importance of the criteria was performed (Table 4). All information concerning the importance of decision makers and individual expert evaluations regarding the rating of the criteria and sub-criteria via attributes and attribute weights usually are expressed in linguistics terms. The set of linguistics terms used to rate the importance of each criteria / sub-criteria for the decision-makers according to standard protocols used during rehabilitation. The ranking by numbers of criteria / sub-criteria linguistics terms is presented in section Results (Table 4, Colum ranking by points).

Table 4. Criteria and sub-criteria ranking according to importance.

**Criteria and sub-criteria**			Percent	Type
	**II. Physical and functional state**		**50 %**	
**c1**	**Pain syndrome**	**Ranking by numbers**	**40 %**	
c1.1	Intensity of the pain	1 point – mild pain; 2 points-moderate pain; 3 points-severe; 4 points-most severe pain	35 %	Min
c1.2	Duration of the pain	1 point – <7 days, 2 points – <4 weeks, 3 points– <3 month, 4 points– <6 month, 5 points > 6 month	20 %	Min
c1.3	Nature of the pain	1 point – intermittent pain, 2 points – dynamic, 3 points – sharp, 4 points – intensive	18 %	Max
c1.4	Dynamic pain	1 points –< 90 points, 2 points – > 90 points	12 %	Max
c1.5	Pain increasing factors	1 point - exercising; 2 points - movements; 3 points - position; 4 points - during rest	10 %	Max
c1.6	Pain decreasing factors	1 point – position, 2 points - movements, 3 points - during rest, 4 points - exercising,	5 %	Min
**c2**	**Shoulder motor function and disability**		**35 %**	
c2.1	Shoulder ROM	1 point - up to (flexion <90, abduction < 90);<br>2 points - over (flexion >95, abduction > 95)	40 %	Max
c2.2	Shoulder muscle strength	1 point – 0, 2 points – 1, 3 points – 3, 4 points - 4 / 5	30 %	Max
c2.3	Shoulder functioning and disability	1 point - 0-5, 2 points - 6-10	15 %	Min

c2.4	Shoulder, arm and hand functioning in ADL	1 point <54, 2 points - 55-68, 3 points - 69-85, 4 points - 86-100	10 %	Min
c2.5	Dexterity of movements	1 point - <69 blocks, 2 points - 70-79 blocks, 3 points - >80 blocks	5 %	Max
**c4**	**Sociodemographic and with injury related characteristics**		**25 %**	
c4.1	Affected muscles	1 point – single affected muscle, 2 points – two affected muscle, 3 points – > two affected muscles	40 %	Min
c4.2	Time since surgery	1 point - <6 days, 2 points - <2 weeks, 3 points - <4 weeks, 4 points - >5 weeks	30 %	Min
c4.3	Age	1 point – 55-60; 2 points – 61-75; 3 points > 76-85	15 %	Min
c4.4	Causes of injury	1 point - Trauma 2 points - Degenerative	10 %	Min
c4.5	Affected arm	1 point - Dominated, 2 points - Not dominated	5 %	Min
	**II. Psychoemocional status**		**30%**	
**c3**	Anxiety / Depression	1 point 0-7, 2 points - 8-10, 3 points 11-14, 4 points 15-21	35 %	Min
**c5**	Fear of movements	1 point - 1-36; 2 points - 37-68	20 %	Min
**c6**	Sleep restriction	1 point - normal sleep, 2 points -sleep restriction first week after surgery, 3 points -sleep restriction during rehabilitation, 4 points -sleep restriction after activities	45 %	Min
**III.**	**IV. Healthcare organization and routine**		**20%**	
**c7**	Rehabilitation measures (Conventional rehabilitation program)		40 %	Max
**c8**	Program intensity and duration (2-2.5 weeks)		20 %	Max
**c9**	Medicines		25 %	Min
**c10**	Home activities and exercising intensity		15 %	Max

3.5. Follow-Up Routine

The conventional rehabilitation program following RC repair consists of measures: physical therapy, occupational therapy, massage, physiotherapy, psychologist, social worker and patient education. Duration of rehabilitation program 2-2.5 weeks with intensity 5 times/week and 3.5-4 hours/day according to an individually prepared rehabilitation protocol with rest breaks between sessions. At the end of rehabilitation, patients were given a home program with an individual home program and physical activity recommendations with individually designed physical load, intensity and duration.

The evaluation of the Physical and functional state of the patient was performed at the beginning (I assessment) and the end (II assessment) of outpatient rehabilitation. The assessment measures were the following: (1) Patients' sociodemographic characteristics and information related to RC pathology, rehabilitation measures, medicines and exercise intensity at home were recorded from

the patient’s medical history. (2) Pain syndrome - intensity, severity, and nature of pain were evaluated by VAS scale [12, 18] and McGill pain questionnaire [19, 22]. (3) Shoulder motor function was evaluated using manual muscle strength testing (MMT) [22-24], shoulder active ROM [25, 27, 28], and dexterity of movements by Box&Block test [26]. (4) Functioning and disability of the Arm, Shoulder and Hand by DASH [27, 29, 30] and SPADI [31]; (5) Psycho-emotional state – anxiety and depression evaluated by HAD scale [14, 19, 30-33], Sleep restrictions [17, 19] and Fear of movements by TAMPA Scale of Kinesiophobia [25, 34, 35].

After ranking, the classification of patients into 5 classes was made using two methods: a heuristic evaluation method based on expert opinion and a classical evaluation method using the K-Means clustering algorithm. After performing the calibration of the comparison of the classification of patients into classes by the two methods, the exact limits of each class are determined, and the differences between the class distributions are evaluated. The heuristic method of grouping patients into classes (based on expert opinion on patient recovery success and rehabilitation outcomes) was based on the methodology of the International Classification of Functioning, Disability and Health (ICF), using a qualification system to assess the impairment of body functions, activity, limitations and participation restrictions in patients, those with shoulder disorders [36, 37]. The final grouping of patients into classes according to rehabilitation outcomes is described as follows:

I class - Complete impairment - a problem that is present more than 95% of the time, with an intensity, which is totally disrupting the persons daily life every day over the last 30 days;

II class - Severe impairment - a problem that is present more than 50% of the time, with an intensity which is partially disrupting the persons daily life over the last 30 days;

III class - Moderate impairment - a problem that is present less than 50% of the time, with an intensity, which is interfering in the persons daily life over the last 30 days;

IV class - Mild impairment - a problem that is present less than 25% of the time, with an intensity a person can tolerate and which rarely happens over the last 30 days;

V class - No impairment - the person has no problem.

The second method of grouping patients into classes was performed using a standard qualitative assessment, applying one of the MCDM methods - the SWARA methodology to determine the weights of the criteria.

3.6. MCDM methods

MCDM methods are generally used for a health care decision-making problem: (1) nature of the decision/goal (sorting, ranking, and choice); (2) criteria type (qualitative/quantitative); (3) criteria structure (flat/hierarchical); (4) weight of criteria (subjective/objective); (5) alternatives (incremental/non-incremental) [7, 11, 38]. MCDM methods mainly include three parts: planning and design, evaluation and selection, and data processing. Data processing focuses on the analysis and identification of rehabilitation management problems and data features to address practical cases. The application gives a ranking result based on the selected criteria, their corresponding values, and assigned weights.

MCDM can be used in rehabilitation in a variety of ways. For example, it can be used to prioritize treatments based on the patient's individual needs and goals [9, 10]. It can also be used to evaluate the effectiveness of different treatment options by comparing the outcomes of each option against a set of predetermined criteria. One common MCDM approach used in rehabilitation is the Analytic Hierarchy Process (AHP) [39, 40]. AHP involves breaking down a decision into a hierarchy of criteria and sub-criteria and then assigning weights to each criterion based on its relative importance. The weights are determined through a pairwise comparison process in which the decision maker compares each criterion to every other criterion and assigns a score based on their relative importance. These scores are then used to

calculate the overall weight of each criterion in the decision. Another MCDM approach that can be used in rehabilitation is the Technique for Order of Preference by Similarity to the Ideal Solution (TOPSIS), which involves ranking different treatment options based on their similarity to an ideal solution [11, 41]. The ideal solution is determined based on a set of predetermined criteria, and the treatment options are then ranked based on their distance from this ideal solution.

3.6.1. SWARA method for criteria weighting

Applying the MCDM framework to solve real-life rehabilitation problems, we need initially to assess the different criteria and determine the corresponding criteria weights. The weighting of criteria importance is the key element in MCDM applications [38]. Different methods of criteria evaluation have been used in solving complex MCDM problems: AHP, SWARA, ANP and others [39]. In the proposed methodology, the SWARA (Step-wise Weight Assessment Ratio) technique [40] is used to define the main criteria weights engaging experts that perform criteria ranking by importance in the criteria weighting process. This method is superior to the AHP method due to a lower number of pairwise comparisons [41] and can be used successfully instead of ANP, FARE, and AHP methods [42].

The SWARA technique for the determination of the criteria weights can be described through the subsequent steps:

1. The definition of the criteria set in rehabilitation (Table 3).
2. Arrangement of the criteria by their importance by experts (in the first position are the most important criteria, and in the last – the least important) (Table 4).
3. Evaluation of the comparative importance of average value of the criterion importance s_j, beginning from the second criterion. It is necessary to find how much criterion s_j is more important than the criterion s_{j+1} and do so for each following criterion (Table 5).

4. The calculation of the comparative importance coefficient is as follows (Table 6, 3rd column):

$$k_j = s_j + 1$$

5. Determination of the recalculated weight is as follows (Table 7, 4th column):

$$q_j = \frac{q_{j-1}}{k_j}$$

6. The calculation of the final weights of criteria (Table 8, 5th column):

$$w_j = \frac{q_j}{\sum_{j=1}^{n} q_j},$$

where n is number of the criterion.

Criteria weights were assessed subjectively by expert. They were arranged in order of importance from the most to the least important. Further, these experts compared the flanking criteria pairs. Values of pairwise comparison were presented in Table 5. Table 6 presents calculations by SWARA method. The percentages from Table 4 were applied to the final sub-criteria (Table 6, 1st and 6th column).

Table 5. Evaluation of relative importance in pairs of criteria (in range from 0 to 1).

Experts	Pairwise evaluation of criteria relative importance								
	$c_{1\leftrightarrow 2}$	$c_{2\leftrightarrow 3}$	$c_{3\leftrightarrow 4}$	$c_{4\leftrightarrow 5}$	$c_{5\leftrightarrow 6}$	$c_{6\leftrightarrow 7}$	$c_{7\leftrightarrow 8}$	$c_{8\leftrightarrow 9}$	$c_{9\leftrightarrow 10}$
1	0.55	0.35	0.25	0.88	0.55	0.85	0.50	0.05	0.99
2	0.65	0.60	0.18	0.71	0.20	0.99	0.43	0.15	0.59
3	0.40	0.55	0.65	0.69	0.15	0.45	0.15	0.25	0.60
4	0.55	0.50	0.30	0.55	0.45	0.52	0.25	0.35	0.42
5	0.70	0.70	0.45	0.70	0.50	0.70	0.60	0.49	0.80
6	0.45	0.65	0.10	0.58	0.05	0.90	0.45	0.10	0.79
7	0.50	0.62	0.30	0.66	0.35	0.96	0.35	0.05	0.66
8	0.40	0.40	0.05	0.80	0.10	0.70	0.65	0.25	0.82
9	0.65	0.55	0.10	0.99	0.05	0.69	0.80	0.50	0.60
10	0.65	0.60	0.05	0.89	0.20	0.85	0.15	0.45	0.70
11	0.45	0.25	0.42	0.77	0.15	0.80	0.45	0.03	0.55

12	0.55	0.30	0.05	0.15	0.05	0.70	0.55	0.55	0.75
Average values	0.5417	0.5058	0.2417	0.6975	0.2333	0.7592	0.4442	0.2683	0.6900

Table 6. Criteria and sub-criteria weighting by SWARA method.

**Criteria**	**Average values of comparative importance criteria,** $s_{j \leftrightarrow j+1}$	**Coefficients of comparative importance criteria,** k_j	**Recalculated (intermediate) criteria weights,** q_j	**Final Criteria weights,** w_j	**Final Sub-Criteria weights,** w_j
c1 0.2	–	1.000	1.000	0.3292	c1.1 0.11522<br>c1.2 0.0658<br>c1.3 0.0593<br>c1.4 0.0395<br>c1.5 0.0329<br>c1.6 0.0165
c2 0.175	0.5417	1.5417	0.6486	0.2135	c2.1 0.0854<br>c2.2 0.0641<br>c2.3 0.0320<br>c2.4 0.0214<br>c2.5 0.0107
c3 0.135	0.5058	1.5058	0.4308	0.1418	-
c4 0.125	0.2417	1.2417	0.3469	0.1142	c4.1 0.0457<br>c4.2 0.0343<br>c4.3 0.0171<br>c4.4 0.0114<br>c4.5 0.0057
c5 0.105	0.6975	1.6975	0.2044	0.0673	-
c6 0.06	0.7592	1.7592	0.0942	0.031	-
c7 0.08	0.2333	1.2333	0.1657	0.0545	-
c8 0.05	0.4442	1.4442	0.0652	0.0215	-
c9 0.04	0.2683	1.2683	0.0514	0.0169	-
c10 0.03	0.6900	1.6900	0.0304	0.0100	-
			**3.0376**		

3.6.2. Aggregation method for the decision support system

In the manuscript, the MCDM methodology is proposed for real-life rehabilitation problems. The methodology can help the rehabilitation specialist to make a decision about whether rehabilitation was successful or not in the particular case. Patients are grouped into classes

according to the success of recovery using the heuristic method and the K-Means clustering algorithm after ranking with the weighted aggregation method [43]. This classical weighted aggregation is known as the simple additive weighting method. The weighted aggregation method (WAM) is realized according to the following steps:

- First, we need to construct a situation vector for each patient and add it to the table. In the table, we present values x_{ij} using the initial information that consists of the evaluations of patients in columns j before and after rehabilitation using a set of the corresponding criteria in rows i.

- Second, when the situation vectors of patients are constructed and presented in the tables, the normalization based on the compromise normalization equation [44] is used to normalize the data of patients:

$$r_{ij} = \frac{x_{ij} - \min_i x_{ij}}{\max_i x_{ij} - \min_i x_{ij}} \text{ (For maximized criterion),}$$

$$r_{ij} = \frac{\max_i x_{ij} - x_{ij}}{\max_i x_{ij} - \min_i x_{ij}} \text{ (For minimized criterion).}$$

- Third, the normalized data is processed to realize the classification of patients according to rehabilitation success and make the final decision. The calculations are done by the WAM formula:

$$\text{WAM} = \sum_{j=1}^{n} (w_j \, r_{ij}),$$

where $w_j = (w_1, w_2 \dots w_n)$ is vector of criteria weights

4. Results

The application of the methodological steps (normalization and weighting) in the context of rehabilitation outcomes - the creation of a decision-making matrix for the primary rehabilitation protocol (i.e. the final value for each patient) at the beginning of rehabilitation is presented in

Table A1 (S1 Appendix) and at the end of rehabilitation is presented in Table A2 (S1 Appendix). Table A3-4 (S1 Appendix) represent the normalized weighted decision-making matrix of 20 patients during I and II assessment rehabilitation outcomes. In order to evaluate the changes in the recovery results of the patients and compare them with the condition of a healthy person, we also added the 21st patient to the matrix and presented a healthy person criterion in the Table A1-2 and Table A3-4 s.

Table 7 shows the aggregation of the common criteria weights of 20 patients during the I-II assessment and a detailed analysis of the weights' aggregation of each group of criteria (arithmetic mean after normalization MaxMin). Then in the 21st place is the aggregation of the criteria weights of a healthy person (Table 7).

Table 7. The criteria weight aggregation during I-II assessment.

	**Arithmetic mean after normalization MaxMin c1/c2/c3/c4/c5/c6 criteria groups**		**Arithmetic mean after normalization MaxMin of each criteria group**							
			**c1 criteria group**		**c2 criteria group**		**c4 criteria group**		**c3/c5/c6 criteria groups**	
	**I**	**II**	I	II	I	II	I	II	I	II
1	**0.4712**	**0.7109**	0.5551	0.8278	0.3404	1.0000	0.4943	0.4943	0.4819	0.4819
2	**0.5179**	**0.7959**	0.5981	0.7992	0.4916	0.8320	0.4210	0.4210	0.3871	0.8579
3	**0.3795**	**0.6338**	0.4249	0.8410	0.0840	0.5053	0.3971	0.3971	0.4899	0.3951
4	**0.5312**	**0.8104**	0.5407	0.5605	0.4702	0.8320	0.7914	0.7914	0.3871	1.0000
5	**0.5260**	**0.6288**	0.3729	0.7281	0.5542	0.4947	0.6057	0.6057	0.4899	0.6794
6	**0.3325**	**0.4073**	0.2681	0.4962	0.2489	0.3573	0.4676	0.4676	0.4819	0.4819
7	**0.5099**	**0.6382**	0.4405	0.5035	0.5542	0.7374	0.6057	0.6057	0.3951	0.6794
8	**0.3169**	**0.4590**	0.4303	0.7250	0.1435	0.2733	0.3000	0.3000	0.4154	0.4627
9	**0.4583**	**0.7841**	0.4460	0.6109	0.4107	0.8320	0.6438	0.6438	0.3871	0.8579
10	**0.3753**	**0.6468**	0.3840	0.4033	0.0595	0.5480	0.6095	0.6095	0.3951	1.0000
11	**0.4647**	**0.6341**	0.3543	0.3755	0.2809	0.6320	0.6057	0.6057	0.5846	1.0000
12	**0.4644**	**0.7443**	0.2956	0.5182	0.3649	0.8320	0.6057	0.6057	0.4899	0.8579
13	**0.5605**	**0.6102**	0.4492	0.6121	0.3924	0.4520	0.5733	0.5733	0.9052	0.8579
14	**0.4209**	**0.5159**	0.3633	0.3246	0.3329	0.4520	0.5790	0.5790	0.6129	0.8579
15	**0.4667**	**0.7617**	0.4704	0.8409	0.2809	0.7267	0.6095	0.6095	0.5846	0.6794
16	**0.3609**	**0.4660**	0.2251	0.3611	0.3329	0.2733	0.2228	0.2228	0.5846	1.0000
17	**0.3440**	**0.6912**	0.5300	0.4850	0.1435	0.8320	0.5086	0.5086	0.1976	1.0000
18	**0.5866**	**0.5973**	0.5651	0.5738	0.4702	0.4947	0.7476	0.7476	0.7077	0.6603
19	**0.2935**	**0.4473**	0.5307	0.7567	0.0595	0.0840	0.3200	0.3200	0.0948	0.4819
20	**0.5118**	**0.8179**	0.4127	0.7549	0.3618	0.8320	0.5086	0.5086	0.4899	0.8579
21	**1.0000**	**1.0000**								

Abbreviations: I- First assessment, II- Second assessment; 21 row stands for healthy person

After a detailed analysis of the weighted aggregation results of individual criteria of each patient group before and after rehabilitation, experts were asked to evaluate the rehabilitation result of each patient by dividing each patient into classes. The assignment of patients into the classes, according to the expert's opinion, did not differ significantly from our used patient's classification method (Table 8). After a discussion with experts, it was agreed / decided that our classification of patients into 5 classes meets the requirements and expectations of rehabilitation specialists according to individual evaluation criteria.

Table 8. Distribution of patients in classes according to the opinion of experts.

Assigning patients to a class according to experts' opinion (class)									
**Patients**	**I assessment**	**1 Expert**	**2 Expert**	**3 Expert**	**II assessment**	**1 Expert**	**2 Expert**	**3 Expert**
1	II class	II	III	II	IV class	III	IV	IV
2	III class	III	III	III	IV class	IV	IV	IV
3	II class	II	II	II	III class	III	III	III
4	III class	III	III	III	IV class	IV	IV	IV
5	III class	III	III	III	III class	III	III	III
6	II class	II	II	II	II class	II	II	III
7	III class	III	III	IV	III class	III	III	III
8	II class	II	II	II	II class	II	II	II
9	II class	II	II	III	IV class	IV	IV	IV
10	II class	II	II	II	III class	III	III	III
11	II class	II	III	II	III class	III	III	III
12	II class	II	II	II	IV class	IV	IV	IV
13	III class	III	III	III	III class	III	III	III
14	II class	II	II	II	III class	III	III	III
15	II class	II	III	II	IV class	IV	IV	IV
16	II class	II	II	II	II class	III	III	II
17	II class	II	II	II	IV class	III	IV	IV
18	III class	III	IV	III	III class	III	III	III
19	II class	III	III	III	II class	III	III	III
20	III class	III	III	III	IV class	IV	IV	IV

The final result of our decision support methodology shows that at the beginning of rehabilitation 90% of patients had severe and moderate impairment and functional problems in daily life activities and were assigned to II-III class (Table 9). The effectiveness of rehabilitation is indisputable, and at the end of rehabilitation was detected a significant improvement in shoulder

motor function, a decrease in pain and reduction in disability. 80% of patients have moderate and mild impairment and were assigned to III and IV classes (Table 9).

Table 9. Classification of patients into classes according to rehabilitation success.

**Classification of patients into classes**		**I class**	**II class**	**III class**	**IV class**	**V class**
		0.0-0.29	0.3-0.49	0.5-0.69	0.7-0.89	0.9-0.99
**Patient's distribution between classes n (%)**	**I assessment**	0 (0 %)	13 (65%)	7 (35%)	0 (0 %)	0 (0 %)
	**II assessment**	0 (0 %)	4 (20%)	8 (40%)	8 (40 %)	0 (0 %)

After the expert assessment, evaluation and verification of the class boundary values were performed using the K-Means clustering method. K-Means clustering is a classic unsupervised learning algorithm that can be applied to grouping diverse data, such as patient data, based on their health status [45]. K-Means calculations are performed as follows:

1. The number of clusters K (in our case is five);
2. Cluster centroids c_i are initialized by k-means++ algorithm [46];
3. $D = d(x_i, c_i)$ - the Euclidean distance is calculated between the cluster centroids and each data point;
4. According to the calculated D, all data points are assigned to the nearest centroid;
5. All cluster centroid positions c_i are recalculated by computing the mean of currently assigned data points:

$$c_i = 1/|C_i| \cdot \sum_{x \in C_i} x_i$$

6. The cycle from the third step is repeated until the position of the cluster centroids no longer changes.

The larger the sample of different patients, the more rehabilitation values we have and the more precisely we can determine the cut-off values for the classes. Therefore, we combine expert assessment before and after rehabilitation. In each case, we have 21 rehabilitation assessments of patients. For the 5 classes of patients, min and max values (intervals) according to experts are presented in Table 9 in the 2nd column. There is one outlier in the expert assessment (value **0.2935)** that is rejected.

After grouping with the K-Means clustering algorithm of the pre-and post-rehabilitation patient values acquired after MCDM processing, we obtain intervals for the fifth classes (Table 9, column 3). The range of values for patients before and after rehabilitation after WAM calculations was [0.2935-1]. We included an additional min value of 0.01 in this range of values. We assumed that if we have a maximum possible value of 1, we should also have a minimum possible value of about 0). Finally, using K-Means clustering for the corrected interval of values (Table 9, column 4), we got rehabilitation ranges of values for the five classes that coincide with the expert evaluation, except for class 1.

In order to avoid gaps between class intervals, we adjust the min and max values of the intervals as follows:

1. From each min value of the following class interval, we subtract the max value of the preceding class interval and divide the difference in half;
2. Further, we add the obtained value to the max value of the lower class and subtract it from the min value of the higher class.

Table 10. Evaluation of the patient class boundary values based on experts and the K-Means clustering method.

Number of classes	Boundaries of classes by experts	Boundaries of classes by K-Means for corrected interval values
**1**	[0.0100 - 0.3168]	[0.0100 - 0.2934]
**2**	[0.3169 - 0.491]	[0.2935 - 0.491]

**3**	[0.492 - 0.668]	[0.492 - 0.668]
**4**	[0.6692 - 0.8179]	[0.669 - 0.8179]
**5**	[0.818 - 1]	[0.818 - 1]

Looking at the results in Table 10, we see that the heuristic evaluation method based on expert opinion and the standard qualitative evaluation methods shows that the intervals for all five classes are similar and do not significantly differ. It is important to note that the applicability of MCDM methods in the field of rehabilitation is acceptable and reliable, and the use of a decision support system would greatly facilitate the work of rehabilitation professionals.

Discussion

The proposed methodology was applied in a case study in the rehabilitation protocol selection after RC tears. The novelty of our research is the development and testing of a new decision support system that combines many factors into a single valuation and allows each patient to assess his capabilities separately according to individual limitations of physical, functional and psycho-emotional functions. The aggregation of the multiple criteria and detailed analysis of factors influencing recovery help to determine and decide on a successful rehabilitation protocol for patients with RC tears.

The reader may naturally wonder why current traditional rehabilitation methods are not suitable. They are appropriate. The application of traditional assessment methods used in rehabilitation is old and reliable and proven by many studies and results of rehabilitation effectiveness. However, the introduction of innovations and the search for more accurate methods are necessary to unify the assessment strategy of specialists, find a common language and terminology, and create a system/algorithm based on mathematical calculations, which would eliminate the subjective (assessor's) approach. Therefore, our case study experts - who have many years of experience in rehabilitation with such types of patients, selected the factors most influencing the recovery of

functions, evaluated the patients and repeated the evaluation after rehabilitation. The selection of criteria was based on the results of recent scientific research and the long-term rehabilitation experience of experts in restoring impaired biopsychosocial functions of patients after RC tears. During the expert's discussion, the criteria and sub-criteria were defined, and after being ranked by importance, their weighting coefficients were calculated, and data processing was performed using multiple-criteria decision-making methods. MCDM methods calculated the results according to the applied algorithms, and the experts were asked again to check and review whether the mathematical methods correctly presented the data and grouped the patients into classes. Due to the detailed weight aggregation results (Table 6-7) of individual criteria for each patient group, it is possible to individually assess which function has the most significant recovery during rehabilitation, which physical or functional problems still lead to impairment and disability, or have led to a worsening of the situation. Based on the received data, the specialist can adjust and analyze the individual rehabilitation protocol: how the applied measures influence the rehabilitation results, what primary factors (age, injury or damaged muscle) determine the choice of the protocol, what factors had the greatest therapeutic effect, what is the patients' physical tolerance and functional recovery. It was also assessed whether the groups formed by experts and those proposed by statistical methods differed significantly. Our study showed that a very similar result was obtained after conducting a heuristic evaluation and applying standard methods. There was not much difference in the distribution of patients into classes, and the class boundaries were similar, which shows us that the MCDM methods are accurate and close and can even be said to correspond to the experience of rehabilitation specialists. This allows us to assume that MCDM methods are sensitive and flexible, allowing for variation according to different pathologies and individualized interventions and programs. However, the most important thing is that this method allows you to simultaneously see the general picture of the patient and accurately record the changes, as well as monitor the progress

or analyze in detail which factor at which time influenced the healing. And thus, it is possible to detail the influencing factors and adjust and apply the rehabilitation program to each specific situation. Evaluation of rehabilitation results and the general physical and functional condition of patients before and after the rehabilitation program was carried out using a multi-criteria support system. The values of each patient's criteria influencing recovery and overall progress were analyzed, the expertise performed, and the results obtained were calibrated with the data of statistical methods, the obtained results of which showed only insignificant differences between assigning criteria weights and classifying patients into classes. This shows the possibility of carrying out a detailed analysis of the criteria/factors influencing the situation of each patient individually, taking into account his condition, rehabilitation time or healing process.

The novelty of MCDM methods lies in their continuous evolution and development, with new approaches and techniques being proposed and improved to better handle increasingly complex decision-making tasks too. In rehabilitation, this allows for a wide range of choices and a flexible decision-making algorithm when analyzing or correcting the rehabilitation protocol.

In our results, we only see the advantages of the methods we used, so we recommend using decision-making systems in rehabilitation in different areas so that we can see the most suitable methods, algorithms, and protocols. Also, in order to determine the limitations of decision-making systems in rehabilitation, we must monitor and continuously analyze the use of these systems in practice for many years.

Conclusions

After the case study using the MCDM methodology, we can see that it perfectly contributes to the decision support system, thus improving the efficiency of parameters' selection and allowing young inexperienced specialists to manage the rehabilitation process more easily. Our results

confirmed that the decision support system could be perfectly applied in rehabilitation when selecting protocols, analyzing different factors influencing rehabilitation outcomes, and predicting the patient's recovery limits.

In summary, firstly, the multi-criteria support system allows us to evaluate the effectiveness of rehabilitation and to monitor changes in the general physical and functional condition of patients during rehabilitation. Secondly, according to the result of the created support system, it is possible to assess the condition or the course of recovery according to the criteria values of individual patients. Finally, the similar evaluation results obtained between expert opinion and statistical methods show an excellent opportunity to analyze each criterion separately, detailing and individualizing according to different pathology, health status or recovery perspective.

References

[1] Keener JD, Galatz LM, Stobbs-Cucchi G, Patton R, Yamaguchi K. Rehabilitation following arthroscopic rotator cuff repair: a prospective randomized trial of immobilization compared with early motion. *J Bone Joint Surg Am*. 2014;96(1):11-19. doi:10.2106/JBJS.M.00034

[2] Saito K, Kenmoku T, Hirota K, Matsui H. Long-term prognoses of patients with and without re-rupture after arthroscopic rotator cuff repair. *J Phys Ther Sci*. 2021;33(6):460-465. doi:10.1589/jpts.33.

[3] Nikolaidou O, Migkou S, Karampalis C. Rehabilitation after Rotator Cuff Repair. Open Orthop J. 2017; 11:154-162. Published 2017 Feb 28. doi:10.2174/1874325001711010154

[4] Berton A, De Salvatore S, Candela V, Cortina G, Lo Presti D, Massaroni C, Petrillo S, Denaro V. Delayed Rehabilitation Protocol after Rotator Cuff Repair. *Osteology*. 2021; 1(1):29-38.

[5] Fahy K, Galvin R, Lewis J, Mc Creesh K. Exercise as effective as surgery in improving quality of life, disability, and pain for large to massive rotator cuff tears: A systematic review & meta-analysis. *Musculoskelet Sci Pract*. 2022; 61:102597. doi:10.1016/j.msksp.2022.102597

[6] Seida JC, LeBlanc C, Schouten JR, et al. Systematic Review: Nonoperative and Operative Treatments for Rotator Cuff Tears. *Ann Intern Med*. 2010;153(4):246-255. doi:10.7326/0003-4819-153-4-201008170-00263

[7] Khan I, Pintelon L, Martin H. The Application of Multicriteria Decision Analysis Methods in Health Care: A Literature Review. *Med Decis Making*. 2022;42(2):262-274. doi:10.1177/0272989X211019040

[8] Cahill PT, Reitzel M, Anaby DR, et al. Supporting rehabilitation stakeholders in making service delivery decisions: a rapid review of multi-criteria decision analysis methods. *Disabil Rehabil*. June 2022:1-14. doi:10.1080/09638288.2022.2080285

[9] Glaize A, Duenas A, Di Martinelly C, Fagnot I. Healthcare decision-making applications using multicriteria decision analysis: A scoping review. *J Multi-Criteria Decis Anal*. 2019;26(1-2):62-83. doi:https://doi.org/10.1002/mcda.1659

[10] Mühlbacher AC, Kaczynski A. Making Good Decisions in Healthcare with Multi-Criteria Decision Analysis: The Use, Current Research and Future Development of MCDA. *Appl Health Econ Health Policy*. 2016;14(1):29-40. doi:10.1007/s40258-015-0203-4

[11] Zeleny M. Compromise programming, Multiple Criteria Decision Making, University of South Carolina Press, 1973, 262–301.

[12] Jain NB, Ayers GD, Fan R, et al. Predictors of pain and functional outcomes after operative treatment for rotator cuff tears. J Shoulder Elbow Surg. 2018;27(8):1393-1400. doi:10.1016/j.jse.2018.04.016

[13] Tangtiphaiboontana J, Figoni AM, Luke A, Zhang AL, Feeley BT, Ma CB. The effects of nonsteroidal anti-inflammatory medications after rotator cuff surgery: a randomized, double-blind, placebo-controlled trial. *J Shoulder Elbow Surg*. 2021;30(9):1990-1997. doi:10.1016/j.jse.2021.05.018

[14] Brochin RL, Zastrow R, Hussey-Andersen L, Parsons BO, Cagle PJ. Revision rotator cuff repair: a systematic review. *J Shoulder Elbow Surg*. 2020;29(3):624-633. doi:10.1016/j.jse.2019.06.023

[15] Abdelwahab A, Ahuja N, Iyengar KP, Jain VK, Bakti N, Singh B. Traumatic rotator cuff tears - Current concepts in diagnosis and management. *J Clin Orthop Trauma*. 2021; 18:51-55. Published 2021 Apr 17.

[16] Lee BG, Cho NS, Rhee YG. Effect of two rehabilitation protocols on range of motion and healing rates after arthroscopic rotator cuff repair: aggressive versus limited early passive exercises. *Arthroscopy*. 2012;28(1):34-42. doi:10.1016/j.arthro.2011.07.012

[17] Kunze KN, Movasagghi K, Rossi DM, et al. Systematic Review of Sleep Quality Before and After Arthroscopic Rotator Cuff Repair: Are Improvements Experienced and Maintained? *Orthop J Sports Med*. 2020;8(12):2325967120969224. Published 2020 Dec 29. doi:10.1177/2325967120969224

[18] Yi D, Lim H, Yim J. Effect of Microcurrent Stimulation on Pain, Shoulder Function, and Grip Strength in Early Post-Operative Phase after Rotator Cuff Repair. *Medicina*. 2021; 57(5):491. https://doi.org/10.3390/medicina57050491

[19] Wong WK, Li MY, Yung PS, Leong HT. The effect of psychological factors on pain, function and quality of life in patients with rotator cuff tendinopathy: A systematic review. *Musculoskelet Sci Pract*. 2020; 47:102173. doi:10.1016/j.msksp.2020.102173

[20] Sun Z, Fu W, Tang X, Chen G, Li J. Systematic review and Meta-analysis on acromioplasty in arthroscopic repair of full-thickness rotator cuff tears. *Acta Orthop Belg*. 2018;84(1):54-61

[21] Wani Z, Abdulla M, Habeebullah A, Kalogriantis S. Rotator cuff tears: Review of epidemiology, clinical assessment and operative treatment. Trauma. 2016;18(3):190-204. doi:10.1177/1460408615596770

[22] Singh Jagdev B, McGrath J, Cole A, Gomaa AR, Chong HH, Singh HP. Total shoulder arthroplasty vs. hemiarthroplasty in patients with primary glenohumeral arthritis with intact rotator cuff: meta-analysis using the ratio of means. *J Shoulder Elbow Surg*. 2022;31(12):2657-2670. doi:10.1016/j.jse.2022.07.012

[23] Altintas B, Anderson NL, Pitta R, et al. Repair of Rotator Cuff Tears in the Elderly: Does It Make Sense? A Systematic Review. *Am J Sports Med*. 2020;48(3):744-753. doi:10.1177/0363546519834574

[24] Kim B-S, Kim B-S. The effect of static and dynamic muscle stretching as part of warm-up procedures on stretching on isokinetic strength, functional movement screen, flexibility and agility in Korean national foil fencers. *Int J Appl Sport Sci*. 2021;33(2):197-205. doi:10.24985/ijass.2021.33.2.197

[25] Wang H, Hu F, Lyu X, et al. Kinesiophobia could affect shoulder function after repair of rotator cuff tears. *BMC Musculoskelet Disord*. 2022;23(1):714. Published 2022 Jul 26. doi:10.1186/s12891-022-05679-x

[26] Hebert JS, Lewicke J, Williams TR, Vette AH. Normative data for modified Box and Blocks test measuring upper-limb function via motion capture. *J Rehabil Res Dev*. 2014;51(6):918-932. doi:10.1682/JRRD.2013.10.0228

[27] Kennedy P, Joshi R, Dhawan A. The Effect of Psychosocial Factors on Outcomes in Patients with Rotator Cuff Tears: A Systematic Review. *Arthroscopy*. 2019;35(9):2698-2706. doi:10.1016/j.arthro.2019.03.043

[28] Hajivandi S, Dachek A, Salimi A, et al. Comparison of the Separate and Combined Effects of Physiotherapy Treatment and Corticosteroid Injection on the Range of Motion and Pain in Nontraumatic Rotator Cuff Tear: A Randomized Controlled Trial. *Adv Orthop*. 2021; 2021:6789453. 2021 Oct 25. doi:10.1155/2021/6789453

[29] Daghiani M, Negahban H, Ebrahimzadeh MH, et al. The effectiveness of comprehensive physiotherapy compared with corticosteroid injection on pain, disability, treatment effectiveness, and quality of life in patients with subacromial pain syndrome: a parallel, single-blind, randomized controlled trial [published online ahead of print, 2022 Mar 5]. *Physiother Theory Pract*. 2022;1-15. doi:10.1080/09593985.2022.2044421

[30] Audigé L, Aghlmandi S, Grobet C, et al. Prediction of Shoulder Stiffness After Arthroscopic Rotator Cuff Repair. The American Journal of Sports Medicine. 2021;49(11):3030-3039. doi:10.1177/03635465211028980

[31] Park JH, Rhee SM, Kim HS, Oh JH. Effects of Anxiety and Depression Measured via the Hospital Anxiety and Depression Scale on Early Pain and Range of Motion After Rotator Cuff Repair. *Am J Sports Med*. 2021;49(2):314-320. doi:10.1177/0363546520976574

[32] Adomavičienė A, Daunoravičienė K, Šidlauskaitė R, et al. Analysis of Functional Recovery and Subjective Well-Being after Arthroscopic Rotator Cuff Repair. Medicina (Kaunas). 2021;57(7):715. Published 2021 Jul 15. doi:10.3390/medicina57070715

[33] Inderhaug E, Kalsvik M, Kollevold KH, Hegna J, Solheim E. Long-term results after surgical treatment of subacromial pain syndrome with or without rotator cuff tear. *J Orthop*. 2018;15(3):757-760. Published 2018 Mar 17. doi:10.1016/j.jor.2018.03.004

[34] Oh LS, Wolf BR, Hall MP, Levy BA, Marx RG. Indications for rotator cuff repair: a systematic review. *Clin Orthop Relat Res*. 2007; 455:52-63. doi:10.1097/BLO.0b013e31802fc175

[35] Wu XL, Briggs L, Murrell GA. Intraoperative determinants of rotator cuff repair integrity: an analysis of 500 consecutive repairs. *Am J Sports Med*. 2012;40(12):2771-2776. doi:10.1177/0363546512462677

[36] Roe Y, Soberg HL, Bautz-Holter E, Ostensjo S. A systematic review of measures of shoulder pain and functioning using the International classification of functioning, disability and health (ICF). BMC musculoskeletal disorders. 2013 Dec;14(1):1-2

[37] Helene L. Soberg, Icon, Kaia B. Engebretsen, Icon,Niels G. Juel, Yngve Roe. Associations between shoulder pain and functioning on the ICF checklist and the disabilities of the arm, shoulder, and hand scale – a cross-sectional study. Disability and Rehabilitation, 2020 May; 42 (21): 3084-3091

[38] Pamučar D, Stević Ž, Sremac S. A New Model for Determining Weight Coefficients of Criteria in MCDM Models: Full Consistency Method (FUCOM). *Symmetry*. 2018; 10(9):393. https://doi.org/10.3390/sym10090393

[39] Odu GO. Weighting Methods for Multi-Criteria Decision Making Technique. *J. Appl. Sci. Environ. Manage*, 2019; 23 (8):1449-1457. doi:10.4314/jasem.v23i8.7

[40] Keršuliene V, Zavadskas EK, Turskis Z. Selection of rational dispute resolution method by applying new step- wise weight assessment ratio analysis (Swara). *J Bus Econ Manag*. 2010;11(2):243-258. doi:10.3846/jbem.2010.12

[41] Stanujkic D, Karabasevic D, Zavadskas EK. A framework for the selection of a packaging design based on the SWARA method. Engineering Economics. 2015 Apr 29;26(2):181-7

[42] Hashemkhani Zolfani S, Bahrami M. Investment prioritizing in high tech industries based on SWARA-COPRAS approach. Technological and Economic Development of Economy. 2014; 3;20(3):534-53

[43] Congedo PM, Baglivo C. Implementation hypothesis of the Apulia ITACA Protocol at district level–part I: The model. Sustainable Cities and Society. 2021 Jul 1;70:102931

[44] Turskis Z, Bausys R, Smarandache F, Kazakeviciute-Januskeviciene G, Zavadskas EK. M-generalised q-neutrosophic extension of CoCoSo method. *International Journal of Computers, Communications & Control*. 2022; 17(1). DOI:10.15837/ijccc.2022.1.4646

[45] Fuente-Tomas L, Arranz B, Safont G, et al. Classification of patients with bipolar disorder using k-means clustering. Guloksuz S, ed. *PLoS One*. 2019;14(1):e0210314.1. doi:10.1371/journal.pone.0210314

[46] Arthur D, Vassilvitskii S. k-means++: The Advantages of Careful Seeding. SODA '07: Proceedings of the Eighteenth Annual ACM-SIAM Symposium on Discrete Algorithms. 2007;37(3):1027–1035

S1 APPENDIX

Table A1. Rehabilitation protocol decision-making matrix (I assessment, points)

I assessments (at the beginning of rehabilitation)																					
**Criteria**	**Patients**																				
	1	2	3	4	5	6	7	8	9	10	11	12	13	14	15	16	17	18	19	20	**21**
c.4.1.	1	2	2	2	1	2	1	3	2	2	1	1	2	2	2	3	2	1	2	2	**1**
c.4.2.	2	1	2	1	2	2	2	2	1	1	2	2	1	2	1	2	2	1	2	2	**1**
c.4.3.	4	2	4	1	4	3	4	4	2	2	4	4	3	3	2	4	4	3	4	4	**1**
c.4.4.	1	2	1	1	1	1	1	1	2	1	1	1	2	1	1	2	1	1	2	1	**1**
c.4.5.	2	3	2	2	1	2	1	2	1	3	1	1	1	1	3	1	1	2	1	1	**1**
c.1.1.	1	1	2	1	2	3	3	2	3	2	3	2	3	3	4	3	3	2	2	2	**1**
c.1.2.	2	2	2	1	1	2	2	2	2	1	2	2	1	1	3	2	4	2	3	3	**4**
c.1.3.	4	2	3	2	2	3	2	2	2	3	3	4	1	5	1	5	5	1	1	5	**1**
c.1.4.	3	3	1	3	2	2	2	2	3	4	2	1	2	2	3	2	1	3	2	4	**4**
c.1.5	1	1	1	1	1	1	1	1	1	1	1	1	2	2	1	1	2	1	1	1	**2**
c.1.6.	1	2	1	2	3	4	1	3	2	4	2	3	4	2	2	3	1	2	3	4	**1**
c2.1	1	2	1	1	1	1	1	1	1	1	1	1	1	1	1	1	1	1	1	2	**2**
c.2.2.	4	3	3	4	4	3	4	3	4	3	4	4	3	3	4	3	3	4	3	3	**4**
c.2.3.	2	3	4	3	3	3	3	3	4	3	3	3	2	3	3	3	3	3	3	2	**1**
c.2.4.	1	1	2	1	2	1	2	2	1	1	1	2	2	2	1	2	2	1	1	1	**3**
c.2.5.	2	1	2	1	1	1	1	2	1	2	2	2	1	1	2	1	2	1	2	2	**1**
c3	2	2	1	2	1	2	1	3	2	1	1	1	1	2	1	1	2	2	3	1	**1**
c5	2	2	2	2	2	2	2	1	2	2	2	2	1	1	2	2	2	1	2	2	**1**
c6	1	2	3	2	3	1	4	3	2	4	2	3	2	3	2	2	4	2	3	3	**1**

Table A2. Rehabilitation protocol decision-making matrix (II assessment, points)

I assessments (at the end of rehabilitation)																					
**Criteria**	**Patients**																				
	1	2	3	4	5	6	7	8	9	10	11	12	13	14	15	16	17	18	19	20	**21**
c.4.1.	1	2	2	2	1	2	1	3	2	2	1	1	2	2	2	3	2	1	2	2	**1**
c.4.2.	2	1	2	1	2	2	2	2	1	1	2	2	1	2	1	2	2	1	2	2	**1**
c.4.3.	4	2	4	1	4	3	4	4	2	2	4	4	3	3	2	4	4	3	4	4	**1**
c.4.4.	1	2	1	1	1	1	1	1	2	1	1	1	2	1	1	2	1	1	2	1	**1**
c.4.5.	2	3	2	2	1	2	1	2	1	3	1	1	1	1	3	1	1	2	1	1	**1**
c.1.1.	1	1	1	1	2	2	3	1	2	2	3	2	2	3	1	3	3	1	2	1	**1**
c.1.2.	4	2	4	2	2	2	4	4	4	2	2	4	2	1	4	4	2	2	4	4	**4**
c.1.3.	5	3	3	3	2	3	2	3	3	3	3	4	2	1	2	5	5	2	2	5	**1**
c.1.4.	4	4	3	4	4	2	3	2	2	4	4	4	3	2	3	4	4	3	4	3	**4**
c.1.5	2	2	2	1	2	2	1	2	2	1	1	1	2	2	2	1	2	1	2	2	**2**
c.1.6.	1	1	1	3	1	2	1	2	2	4	2	3	2	4	2	3	1	2	3	1	**1**
c2.1	2	2	2	2	1	1	2	1	2	2	2	2	1	1	2	1	2	1	1	2	**2**
c.2.2.	4	4	3	4	4	3	4	3	4	4	4	4	3	3	4	3	4	4	3	4	**4**
c.2.3.	1	1	1	1	2	2	2	2	1	2	2	1	1	1	1	2	1	2	2	1	**1**
c.2.4.	3	1	2	1	2	3	2	2	1	2	3	1	2	2	2	2	1	2	2	1	**3**
c.2.5.	1	1	2	1	1	1	1	1	1	2	2	1	1	1	2	1	1	1	2	1	**1**
c3	2	1	1	1	1	2	1	3	1	1	1	1	1	1	1	1	1	2	2	1	**1**
c5	2	1	2	1	2	2	2	1	1	1	1	1	1	1	2	1	1	1	2	1	**1**
c6	1	2	3	1	1	1	1	2	2	1	1	2	2	2	1	1	1	2	1	2	**1**

Table A3-4. Weighted decision-making matrix during I-II assessments.

	**Patients (I assessment)**																				
	**1**	**2**	**3**	**4**	**5**	**6**	**7**	**8**	**9**	**10**	**11**	**12**	**13**	**14**	**15**	**16**	**17**	**18**	**19**	**20**	**21**
c.4.1.	0,0229	0,0114	0,0114	0,0114	0,0229	0,0114	0,0229	0,0000	0,0114	0,0114	0,0229	0,0229	0,0114	0,0114	0,0114	0,0000	0,0114	0,0229	0,0114	0,0114	**0,0229**
c.4.2.	0,0000	0,0115	0,0000	0,0115	0,0000	0,0000	0,0000	0,0000	0,0115	0,0115	0,0000	0,0000	0,0115	0,0000	0,0115	0,0000	0,0000	0,0115	0,0000	0,0000	**0,0115**
c.4.3.	0,0000	0,0267	0,0000	0,0400	0,0000	0,0133	0,0000	0,0000	0,0267	0,0267	0,0000	0,0000	0,0133	0,0133	0,0267	0,0000	0,0000	0,0133	0,0000	0,0000	**0,0400**
c.4.4.	0,0172	0,0000	0,0172	0,0172	0,0172	0,0172	0,0172	0,0172	0,0000	0,0172	0,0172	0,0172	0,0000	0,0172	0,0172	0,0000	0,0172	0,0172	0,0000	0,0172	**0,0172**
c.4.5.	0,0257	0,0000	0,0257	0,0257	0,0514	0,0257	0,0514	0,0257	0,0514	0,0000	0,0514	0,0514	0,0514	0,0514	0,0000	0,0514	0,0514	0,0257	0,0514	0,0514	**0,0514**
c.1.1.	0,1210	0,1210	0,0807	0,1210	0,0807	0,0403	0,0403	0,0807	0,0403	0,0807	0,0403	0,0807	0,0403	0,0403	0,0000	0,0403	0,0403	0,0807	0,0807	0,0807	**0,1210**
c.1.2.	0,0217	0,0217	0,0217	0,0000	0,0000	0,0217	0,0217	0,0217	0,0217	0,0000	0,0217	0,0217	0,0000	0,0000	0,0433	0,0217	0,0650	0,0217	0,0433	0,0433	**0,0650**
c.1.3.	0,0163	0,0488	0,0325	0,0488	0,0488	0,0325	0,0488	0,0488	0,0488	0,0325	0,0325	0,0163	0,0650	0,0000	0,0650	0,0000	0,0000	0,0650	0,0650	0,0000	**0,0650**
c.1.4.	0,0258	0,0258	0,0000	0,0258	0,0129	0,0129	0,0129	0,0129	0,0258	0,0387	0,0129	0,0000	0,0129	0,0129	0,0258	0,0129	0,0000	0,0258	0,0129	0,0387	**0,0387**
c.1.5	0,0000	0,0000	0,0000	0,0000	0,0000	0,0000	0,0000	0,0000	0,0000	0,0000	0,0000	0,0000	0,0453	0,0453	0,0000	0,0000	0,0453	0,0000	0,0000	0,0000	**0,0453**
c.1.6.	0,0222	0,0148	0,0222	0,0148	0,0074	0,0000	0,0222	0,0074	0,0148	0,0000	0,0148	0,0074	0,0000	0,0148	0,0148	0,0074	0,0222	0,0148	0,0074	0,0000	**0,0222**
c2.1	0,0000	0,0912	0,0000	0,0000	0,0000	0,0000	0,0000	0,0000	0,0000	0,0000	0,0000	0,0000	0,0000	0,0000	0,0000	0,0000	0,0000	0,0000	0,0000	0,0912	**0,0912**
c.2.2.	0,0698	0,0000	0,0000	0,0698	0,0698	0,0000	0,0698	0,0000	0,0698	0,0000	0,0698	0,0698	0,0000	0,0000	0,0698	0,0000	0,0000	0,0698	0,0000	0,0000	**0,0698**
c.2.3.	0,0181	0,0090	0,0000	0,0090	0,0090	0,0090	0,0090	0,0090	0,0000	0,0090	0,0090	0,0090	0,0181	0,0090	0,0090	0,0090	0,0090	0,0090	0,0090	0,0181	**0,0271**
c.2.4.	0,0000	0,0000	0,0082	0,0000	0,0082	0,0000	0,0082	0,0082	0,0000	0,0000	0,0000	0,0082	0,0082	0,0082	0,0000	0,0082	0,0082	0,0000	0,0000	0,0000	**0,0164**
c.2.5.	0,0000	0,0378	0,0000	0,0378	0,0378	0,0378	0,0378	0,0000	0,0378	0,0000	0,0000	0,0000	0,0378	0,0378	0,0000	0,0378	0,0000	0,0378	0,0000	0,0000	**0,0378**
c3	0,0738	0,0738	0,1476	0,0738	0,1476	0,0738	0,1476	0,0000	0,0738	0,1476	0,1476	0,1476	0,1476	0,0738	0,1476	0,1476	0,0738	0,0738	0,0000	0,1476	**0,1476**
c5	0,0000	0,0000	0,0000	0,0000	0,0000	0,0000	0,0000	0,0731	0,0000	0,0000	0,0000	0,0000	0,0731	0,0731	0,0000	0,0000	0,0000	0,0731	0,0000	0,0000	**0,0731**
c6	0,0368	0,0245	0,0123	0,0245	0,0123	0,0368	0,0000	0,0123	0,0245	0,0000	0,0245	0,0123	0,0245	0,0123	0,0245	0,0245	0,0000	0,0245	0,0123	0,0123	**0,0368**
**Total:**	**0.4712**	**0.5179**	**0.3795**	**0.5312**	**0.5260**	**0.3325**	**0.5099**	**0.3169**	**0.4583**	**0.3753**	**0.4647**	**0.4644**	**0.5605**	**0.4209**	**0.4667**	**0.3609**	**0.3440**	**0.5866**	**0.2935**	**0.5118**	**1.0000**

Criteria	**Patients (II assessment)**																				
	**1**	**2**	**3**	**4**	**5**	**6**	**7**	**8**	**9**	**10**	**11**	**12**	**13**	**14**	**15**	**16**	**17**	**18**	**19**	**20**	**21**
c.4.1.	0,0229	0,0114	0,0114	0,0114	0,0229	0,0114	0,0229	0,0000	0,0114	0,0114	0,0229	0,0229	0,0114	0,0114	0,0114	0,0000	0,0114	0,0229	0,0114	0,0114	**0,0229**
c.4.2.	0,0000	0,0115	0,0000	0,0115	0,0000	0,0000	0,0000	0,0000	0,0115	0,0115	0,0000	0,0000	0,0115	0,0000	0,0115	0,0000	0,0000	0,0115	0,0000	0,0000	**0,0115**
c.4.3.	0,0000	0,0267	0,0000	0,0400	0,0000	0,0133	0,0000	0,0000	0,0267	0,0267	0,0000	0,0000	0,0133	0,0133	0,0267	0,0000	0,0000	0,0133	0,0000	0,0000	**0,0400**
c.4.4.	0,0172	0,0000	0,0172	0,0172	0,0172	0,0172	0,0172	0,0172	0,0000	0,0172	0,0172	0,0172	0,0000	0,0172	0,0172	0,0000	0,0172	0,0172	0,0000	0,0172	**0,0172**
c.4.5.	0,0257	0,0000	0,0257	0,0257	0,0514	0,0257	0,0514	0,0257	0,0514	0,0000	0,0514	0,0514	0,0514	0,0514	0,0000	0,0514	0,0514	0,0257	0,0514	0,0514	**0,0514**
c.1.1.	0,1210	0,1210	0,1210	0,1210	0,0605	0,0605	0,0000	0,1210	0,0605	0,0605	0,0000	0,0605	0,0605	0,0000	0,1210	0,0000	0,0000	0,1210	0,0605	0,1210	**0,1210**
c.1.2.	0,0650	0,0217	0,0650	0,0217	0,0217	0,0217	0,0650	0,0650	0,0650	0,0217	0,0217	0,0650	0,0217	0,0000	0,0650	0,0650	0,0217	0,0217	0,0650	0,0650	**0,0650**
c.1.3.	0,0000	0,0325	0,0325	0,0325	0,0488	0,0325	0,0488	0,0325	0,0325	0,0325	0,0325	0,0163	0,0488	0,0650	0,0488	0,0000	0,0000	0,0488	0,0488	0,0000	**0,0650**
c.1.4.	0,0387	0,0387	0,0193	0,0387	0,0387	0,0000	0,0193	0,0000	0,0000	0,0387	0,0387	0,0387	0,0193	0,0000	0,0193	0,0387	0,0387	0,0193	0,0387	0,0193	**0,0387**
c.1.5	0,0453	0,0453	0,0453	0,0000	0,0453	0,0453	0,0000	0,0453	0,0453	0,0000	0,0000	0,0000	0,0453	0,0453	0,0453	0,0000	0,0453	0,0000	0,0453	0,0453	**0,0453**
c.1.6.	0,0222	0,0222	0,0222	0,0074	0,0222	0,0148	0,0222	0,0148	0,0148	0,0000	0,0148	0,0074	0,0148	0,0000	0,0148	0,0074	0,0222	0,0148	0,0074	0,0222	**0,0222**
c2.1	0,0912	0,0912	0,0912	0,0912	0,0000	0,0000	0,0912	0,0000	0,0912	0,0912	0,0912	0,0912	0,0000	0,0000	0,0912	0,0000	0,0912	0,0000	0,0000	0,0912	**0,0912**
c.2.2.	0,0698	0,0698	0,0000	0,0698	0,0698	0,0000	0,0698	0,0000	0,0698	0,0698	0,0698	0,0698	0,0000	0,0000	0,0698	0,0000	0,0698	0,0698	0,0000	0,0698	**0,0698**
c.2.3.	0,0271	0,0271	0,0271	0,0271	0,0000	0,0000	0,0000	0,0000	0,0271	0,0000	0,0000	0,0271	0,0271	0,0271	0,0271	0,0000	0,0271	0,0000	0,0000	0,0271	**0,0271**
c.2.4.	0,0164	0,0000	0,0082	0,0000	0,0082	0,0164	0,0082	0,0082	0,0000	0,0082	0,0164	0,0000	0,0082	0,0082	0,0082	0,0082	0,0000	0,0082	0,0082	0,0000	**0,0164**
c.2.5.	0,0378	0,0378	0,0000	0,0378	0,0378	0,0378	0,0378	0,0378	0,0378	0,0000	0,0000	0,0378	0,0378	0,0378	0,0000	0,0378	0,0378	0,0378	0,0000	0,0378	**0,0378**
c3	0,0738	0,1476	0,1476	0,1476	0,1476	0,0738	0,1476	0,0000	0,1476	0,1476	0,1476	0,1476	0,1476	0,1476	0,1476	0,1476	0,1476	0,0738	0,0738	0,1476	**0,1476**
c5	0,0000	0,0731	0,0000	0,0731	0,0000	0,0000	0,0000	0,0731	0,0731	0,0731	0,0731	0,0731	0,0731	0,0731	0,0000	0,0731	0,0731	0,0731	0,0000	0,0731	**0,0731**
c6	0,0368	0,0184	0,0000	0,0368	0,0368	0,0368	0,0368	0,0184	0,0184	0,0368	0,0368	0,0184	0,0184	0,0184	0,0368	0,0368	0,0368	0,0184	0,0368	0,0184	**0,0368**
**Total:**	**0.7109**	**0.7959**	**0.6338**	**0.8104**	**0.6288**	**0.4073**	**0.6382**	**0.4590**	**0.7841**	**0.6468**	**0.6341**	**0.7443**	**0.6102**	**0.5159**	**0.7617**	**0.4660**	**0.6912**	**0.5973**	**0.4473**	**0.8179**	**1.0000**

Click here to access/download;Figure;FIG.1.tif.tif

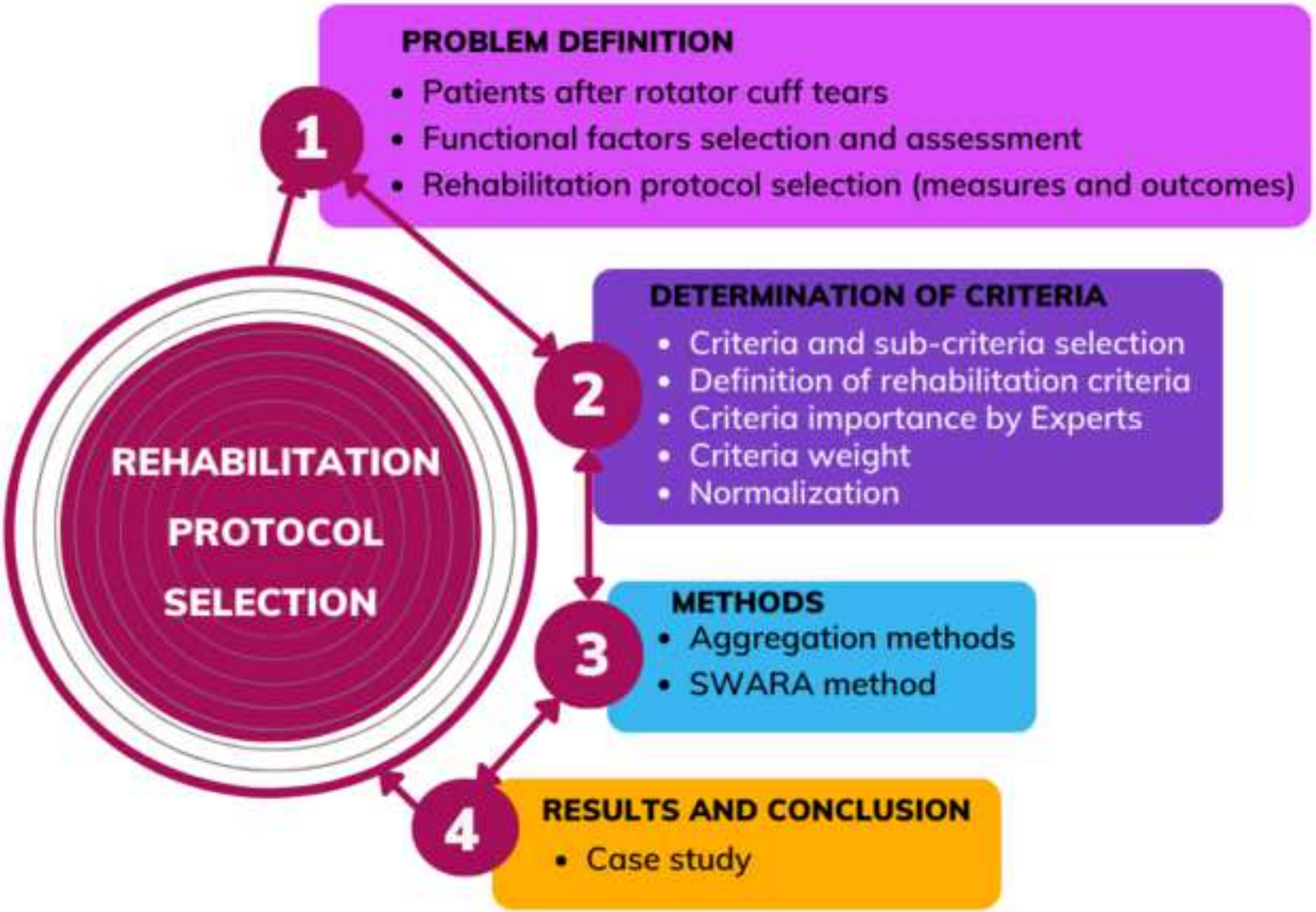

Figure2

Click here to access/download;Figure;FIG.2.tif.tif

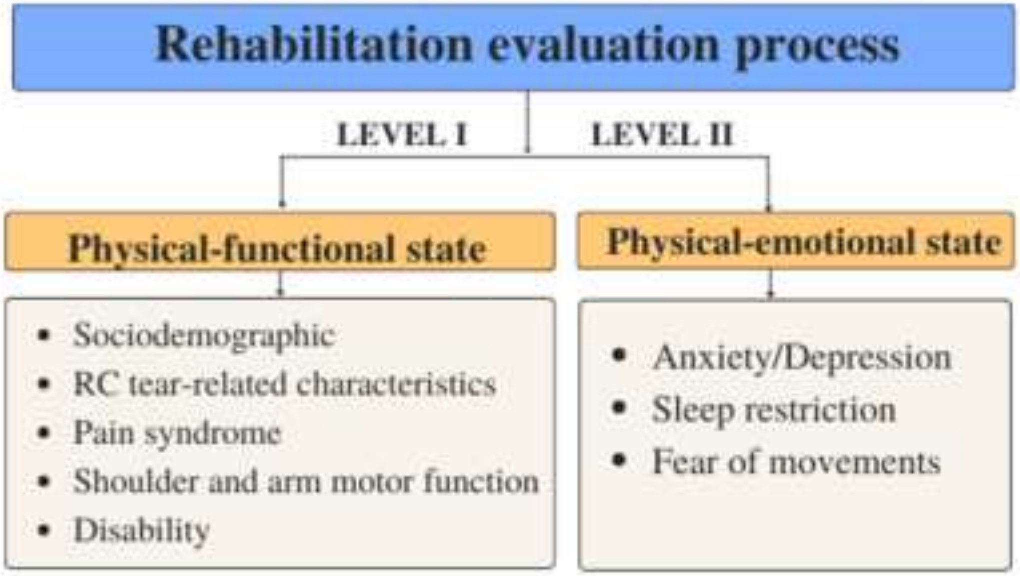

Data set

Click here to access/download
Supporting Information
Criteria_Swara_initialData_normalization_MCDM.xlsx

ANSWER to EDITORS

Reason to Reject PONE – D-23-28958

We recognize that you obtained ethical approval from your local ethics committee after you began the study: your ethics approval document is dated May 25, 2021, while participants were recruited beginning on May 21, 2021. For human subjects research, PLOS ONE requires that authors obtain ethics committee approval before beginning the study, and we do not accept retrospective ethical approval.

We are very sorry that a technical, human error was made when entering the recruitment term. We have corrected and resubmitted the publication and hope for its scientific value.
