# Supplementary material for: Functional recovery prediction during rehabilitation after rotator cuff tears by decision support system
Source: PLoS One. 2024 Mar 25;19(3):e0296984. doi: 10.1371/journal.pone.0296984 (PMC10962824; doi:10.1371/journal.pone.0296984)
Supplement: Attachment — Submitted filename: PLUS ONE.docx [file pone.0296984.s006.docx]

This article investigates Functional recovery prediction during rehabilitation after cuff tears by decision support system. It is an interesting and new topic. The statistical analysis has been performed appropriately and the manuscript written in standard English.

 The comments below may help you rewrite the article.

**Abstract:**

- The method section does not explain the type of study and exact methodology, (including the number of patients, rehabilitation treatment, etc.). It seems better to add these items.

- Nothing has been written about MCDM in the results section.

**Main text**

In the participants’ section:

- where were the rehabilitation treatments done? The name of the center and...

- Why do you insert 24 patients? How was the sample size calculated?

- In line 219, you wrote 6-7 weeks after surgery; however, in line 225, you wrote 5-6 weeks after surgery...it is not clear to the reader….

- Please insert the registration number and URL.

- Please insert the ethical code.

In the follow-up routine section:

- What was the home exercise program? Was it done daily? How were the home exercises checked?

Results section:

- Is it not necessary to write the demographic information in a table? Do demographic characteristics not affect treatment response?

- Maybe it's better to put a study flow diagram.
